# Sliding ferroelectricity in van der Waals layered γ-InSe semiconductor

**DOI:** 10.1038/s41467-022-35490-0

**Published:** 2023-01-03

**Authors:** Fengrui Sui, Min Jin, Yuanyuan Zhang, Ruijuan Qi, Yu-Ning Wu, Rong Huang, Fangyu Yue, Junhao Chu

**Affiliations:** 1 Key Laboratory of Polar Materials and Devices (MOE), School of Physics and Electronic Science, East China Normal University, Shanghai, 200062 China; 2 College of Materials, Shanghai Dianji University, Shanghai, 201306 China; 3 State Key Laboratory of Functional Materials for Informatics, Shanghai Institute of Microsystem and Information Technology, Chinese Academy of Sciences, Shanghai, 200050 China

**Keywords:** Ferroelectrics and multiferroics, Two-dimensional materials

## Abstract

Two-dimensional (2D) van-der-Waals (vdW) layered ferroelectric semiconductors are highly desired for in-memory computing and ferroelectric photovoltaics or detectors. Beneficial from the weak interlayer vdW-force, controlling the structure by interlayer twist/translation or doping is an effective strategy to manipulate the fundamental properties of 2D-vdW semiconductors, which has contributed to the newly-emerging sliding ferroelectricity. Here, we report unconventional room-temperature ferroelectricity, both out-of-plane and in-plane, in vdW-layered γ-InSe semiconductor triggered by yttrium-doping (InSe:Y). We determine an effective piezoelectric constant of ∼7.5 pm/V for InSe:Y flakes with thickness of ∼50 nm, about one order of magnitude larger than earlier reports. We directly visualize the enhanced sliding switchable polarization originating from the fantastic microstructure modifications including the stacking-faults elimination and a subtle rhombohedral distortion due to the intralayer compression and continuous interlayer pre-sliding. Our investigations provide new freedom degrees of structure manipulation for intrinsic properties in 2D-vdW-layered semiconductors to expand ferroelectric candidates for next-generation nanoelectronics.

## Abstract (web summary)

Exploring two-dimensional layered ferroelectric semiconductors is highly desired for ferroelectric-based devices. Here, the authors realize the room-temperature ferroelectricity in layered Y-doped γ-InSe due to the microstructure modifications.

## Introduction

2D vdW-layered ferroelectric semiconductors^1^, with combinations of electric-field switchable polarization and distinctive electronic band structure, offer a wide range of physical opportunities and technological applications in nanoelectronics in the era of information technology, such as in-memory computing and efficient ferroelectric photovotaics^2^. Also, new-principle devices were theoretically predicted in 2D vdW ferroelectric semiconductors, involving the quantum spin Hall effect and even the abnormal valley-related (spin) Hall effect when the valley polarization is further considered (or ferrovalley semiconductors)^3,4^. However, limited to the extremely rare 2D layered parent crystals that belong to the noncentrosymmetric polar space group, only a constrained number of 2D intrinsic ferroelectrics have been successfully demonstrated so far, such as distorted 1 T transition-metal dichalcogenides (TMDs)^5^, In_2_Se_3_^6^, CuInP_2_S_6_^7^ and MX (e.g., GeS)^8^. Continuous efforts have been devoted to exploring for new 2D ferroelectric semiconductors. Owing to the weak interlayer vdW-force in 2D-layered materials, structural control by interlayer twist/translation has successfully enabled the realization of the unexpected interlayer sliding ferroelectricity in Moiré superlattices and TMD heterostructures with artificially-tuned broken inversion symmetry via much more complex fabrication procedures^9–18^. On the other hand, doping, especially with rare-earth elements^19^, which can change the lattice structure (such as bond distance and angle, and atom position) of materials, e.g., via microstrain/structure constrain, has also been employed as an effective strategy to tune ferroelectric properties not only in traditional perovskite-based ferroelectrics^20^ but also in newly-emerging HfO_2_-based ferroelectrics^21,22^, showing great potential for manipulating the fundamental properties of 2D vdW semiconductors.

The 2D vdW-layered InSe semiconductor with suitable bandgap (*E*_*g*_ = 1.0~1.6 eV) and high mobility (~10^3^ cm^2^·V^−1^·S^−1^) has demonstrated intriguing physical and optoelectrical properties^23^. It is theoretically predicted that γ-InSe with *R3m* symmetry may exhibit sliding ferroelectricity^2^; however, no experimental studies on the ferroelectricity, either intrinsic or doping-induced, are available, except for a few reports about the piezoelectricity^24^. This definitely impedes its practical application in nanoferroelectrics. Here, with Y, a member of rare-earth elements that can be considered as “vitamin” dopants, we fantastically improve the crystal quality of vdW-layered γ-InSe prepared by Bridgman method, and realize both robust out-of-plane (OOP) and in-plane (IP) ferroelectricity in InSe:Y at room-temperature. Our atomic imaging results show that Y plays an important role in introducing anisotropic stress/strain in InSe and results in the corresponding microstructure modifications including the stacking-faults elimination and a subtle rhombohedral distortion with intralayer compression along the *c*-direction (i.e., the thickness direction of the 2D layered InSe) and continuous interlayer pre-sliding, which is supposed to enhance both OOP and IP spontaneous polarizations in InSe. Our optical characterizations together with first-principles calculations indicate that the doping-induced structural perturbations slightly broaden the bandgap of intrinsically-direct InSe and significantly optimize the interlayer transport and distribution of intralayer carriers, which reflects the increase of the interlayer stacking order that are likewise beneficial to the enhancement of OOP and IP spontaneous polarizations. Our work provides a strategy to expand the family member of 2D vdW-layered ferroelectric semiconductors.

## Results

### Characterization of structures

**Fig. 1:**
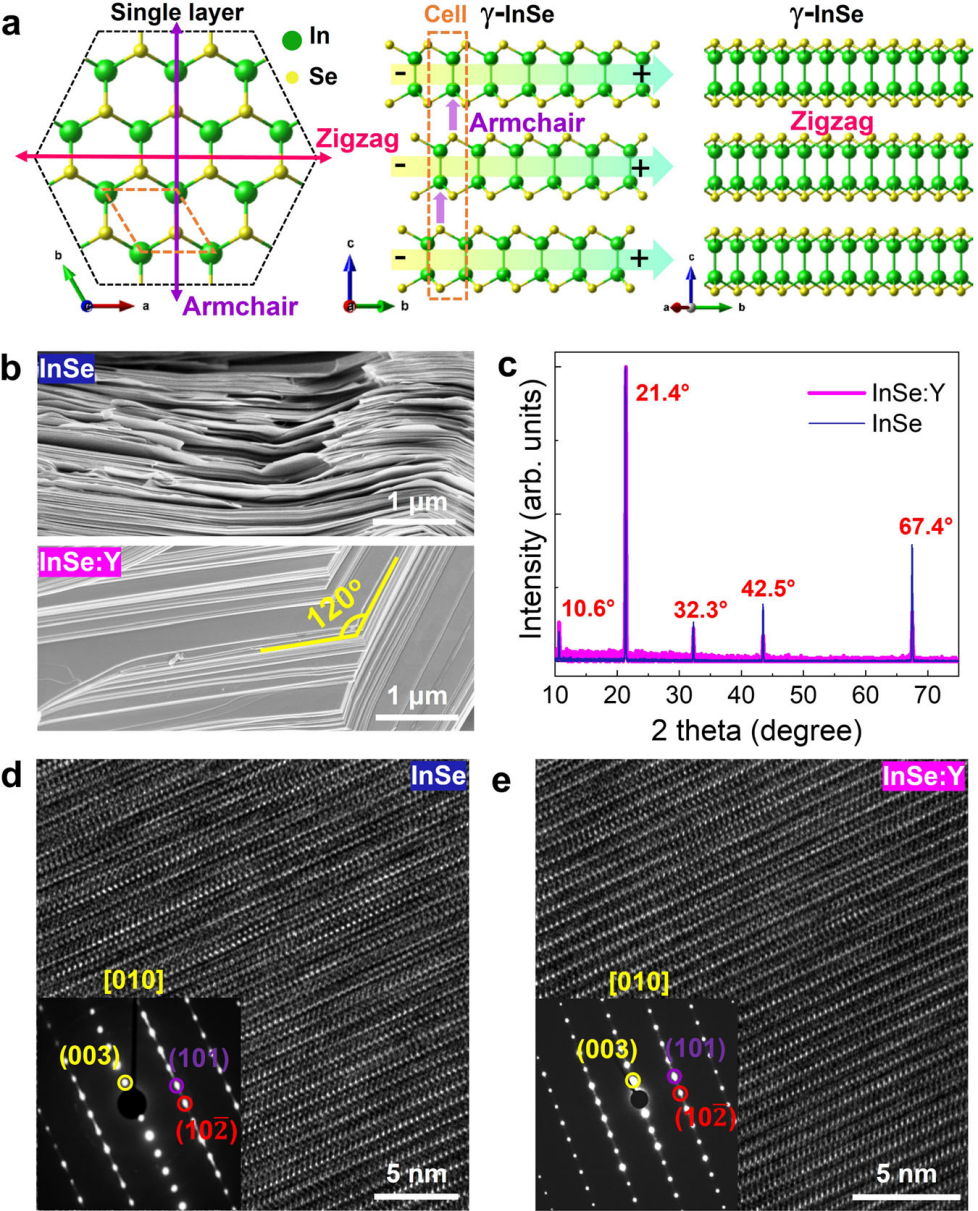
Structural characterization of InSe and InSe:Y. **a** Schematics of InSe single layer and the atomic arrangement of γ-InSe, demonstrating the IP polarization along the AC direction and the OOP polarization along the thickness-direction (see arrows in the middle panel). **b** SEM images of InSe and InSe:Y. **c** XRD patterns of single crystalline InSe and InSe:Y. HRTEM images and the corresponding SAED patterns (inset) of InSe (**d**) and InSe:Y (**e**) projected along the [010] zone axis.

The polymorphic phases of InSe are schematically illustrated in Supplementary Fig. 1, in which γ-InSe is rhombohedral with lattice parameters *a* = *b* = 4.00 Å and *c* = 25.32 Å, and belongs to the *R3m* (*C*^5^_3 v_) noncentrosymmetric space group with ABC-style stacking, possessing three armchair (AC) and three zigzag (ZZ) directions (Fig. 1a)^25^, exhibiting IP and OOP polarizations. Scanning electron microscopy (SEM) gives the morphology of the as-grown InSe and InSe:Y crystals (Fig. 1b and Supplementary Fig. 2a, b). It is worth noting that the undoped InSe presents a soft and plastic lamellar morphology, due to its superplastic nature induced by interlayer gliding and cross-layer dislocation slip^26^, while InSe:Y exhibits a well-defined layer structure with a straight and wrinkle-free surface, indicating the fantastical reversion of the natural plasticity of InSe. Both X-ray diffraction (XRD) patterns of InSe and InSe:Y (Fig. 1c) show obvious {00l} reflections without peak shifts between them, indicating that Y-doping has not changed the single-crystal structure of InSe. Additionally, the Raman spectra of both samples (Supplementary Fig. 2e) display three InSe-related characteristic vibrational modes of *A*^1^_1g_, *E*^1^_2g_ and *A*^2^_1g_. Notice that the actual phase structure of InSe cannot be precisely elucidated based on XRD and Raman characterizations, since all three polytypes of InSe possess similar diffraction peaks and vibrational modes (Supplementary Note 1)^27^. Thus, we employ transmission electron microscopy (TEM) to explore the actual InSe phase. As shown in the cross-section TEM images (Fig. 1d, e and Supplementary Fig. 2c, d) and the corresponding selected area electron diffraction (SAED) patterns (insets in Fig. 1d, e) projected along the [010] zone axis, both samples present a 2D layered structure with obvious electron diffraction features of γ-InSe (Supplementary Fig. 1e), implying that Y-doping does not change the phase of InSe^28^. Energy dispersive spectrometer (EDS) results (both SEM‒EDS and TEM‒EDS; Supplementary Fig. 2f) show the chemical composition of In:Se ≈ 1:1 in InSe and InSe:Y. Due to the high impurity segregation coefficient of InSe^29^ and the low Y-doping level (only 1 at% in the raw material), the actual Y-content is extremely low, which cannot be detected by EDS. We then performed time-of-flight secondary ion mass spectrometry (TOF-SIMS) analysis, proving the uniform distribution of doped-Y with a truly low concentration and a positive valence state (Supplementary Fig. 3). Interestingly, many natural stacking-faults are observed in undoped InSe (Fig. 1d, and Supplementary Fig. 2c) because of the very low formation energy (almost zero) that is responsible for the interlayer glide-induced superplasticity^30^, but are significantly reduced in InSe:Y (Fig. 1e and Supplementary Fig. 2d and Note 1). Based on these TEM results, it can be speculated that trace-Y can effectively inhibit stacking-faults formation, and thus has a striking influence on the crystal microstructure and plasticity of InSe, which may give rise to new physical properties.

### Band structure and carrier transport

**Fig. 2:**
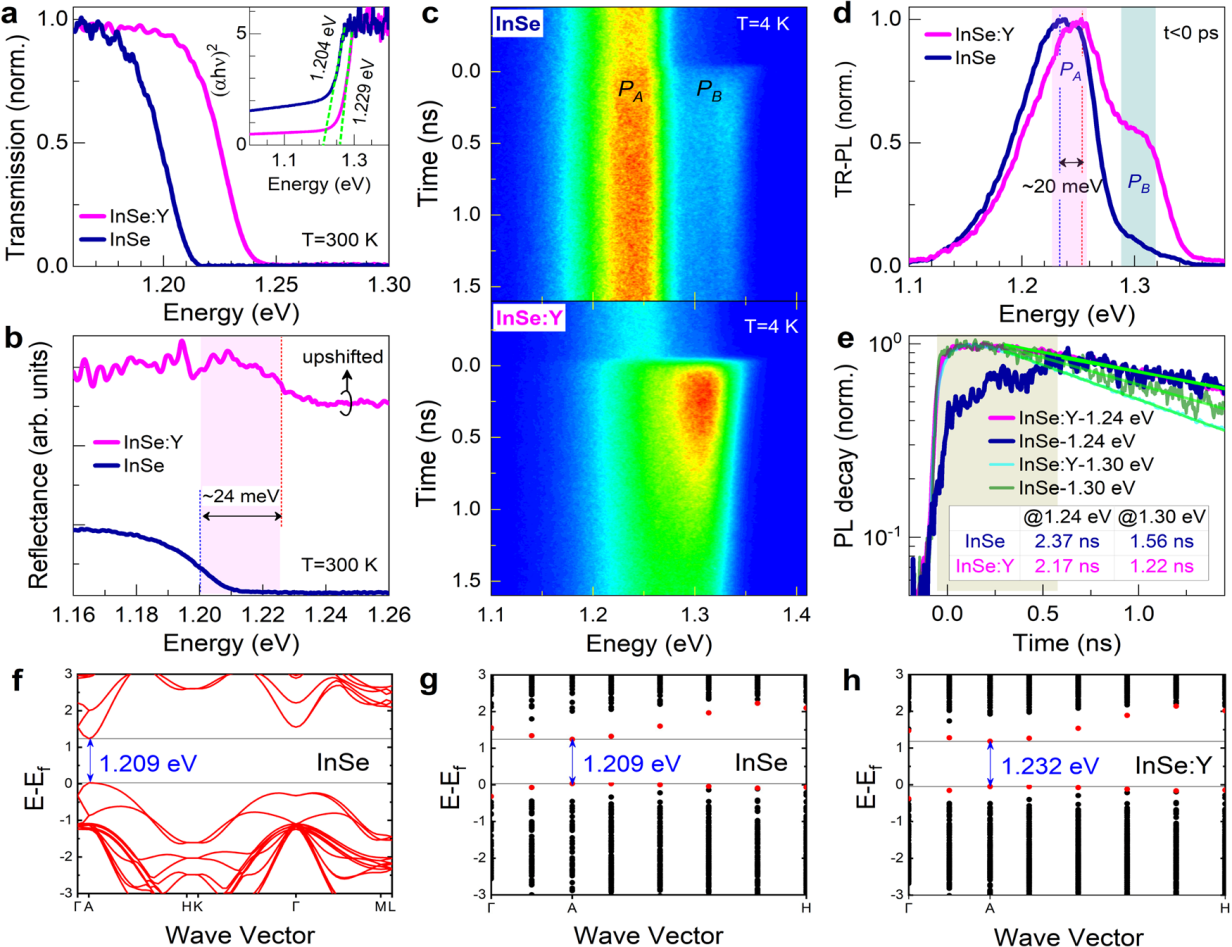
Optical characterizations of InSe and InSe:Y. **a** Transmission spectra at room temperature. The inset gives the corresponding Tauc-plots. **b** Reflectance spectra at room temperature. **c** TR-PL images with peaks *P*_*A*_ and *P*_*B*_ of InSe (upper) and InSe:Y (lower) at 4 K. **d** Transient-PL curves at *t* < 0 ps (i.e., before the next pulse). **e** PL decay curves at ~1240 meV and 1300 meV for both samples. The inset gives the corresponding lifetimes from the fit procedure with a single-exponential decay function. The calculated band structure of InSe (**f**) and the energy eigenvalues for selected k-points near valence band maximum (VBM) based on the supercell of InSe (**g**) and InSe:Y (**h**) (Methods, Supplementary Fig. 5). The bandgap widens with Y-doping, and the VBM stays at the A point. No defect level is introduced in the bandgap by Y-doping.

We perform optical characterizations to investigate the influence of Y-doping-induced microstructure perturbations on the optical properties of InSe. The room temperature transmission spectra (Fig. 2a) of both flakes (InSe and InSe:Y) show a direct transition feature, and the intrinsic absorption edge of InSe:Y has a visible blueshift. Tauc-plots^31^ (inset in Fig. 2a) give *E*_*g*_ ~ 1204 meV for InSe and ~1229 meV for InSe:Y, consistent with the result from the reflectance spectra (Fig. 2b). We perform time-resolved photoluminescence (TR-PL) measurements (Fig. 2c) to further visualize the carrier transition and recombination dynamics. Both crystals present a double-peak structure, of which the low-energy peak (*P*_A_) is related to the intralayer exciton recombination (i.e., the conventional *E*_*g*_) while the high-energy peak (*P*_B_) can be ascribed to the interlayer exciton recombination by considering the slight energy separation of ~60 meV from *P*_A_ and a close lifetime to that of *P*_A_ (Supplementary Note 2)^32,33^. Similarly, *P*_A_ in InSe:Y shows a blueshift of ~20 meV (at 4 K), as observed in transmission and reflectance spectra. More importantly, we should emphasize that i) the intensity of *P*_B_ (interlayer) is largely enhanced by Y-doping as compared to that of *P*_A_ (intralayer) (Fig. 2c, d); ii) the intralayer nonequilibrium carriers (*P*_A_) in InSe:Y have a sharp rise, but are substantially delayed by ~630 ps in InSe, although the lifetime constant of both crystals is subequal within 1 − 3 ns (Fig. 2e); and iii) the spatially-dependent TR-PL results give uniform peak energy and lifetime for InSe:Y, but relatively broad distributions of lifetime for InSe (Supplementary Fig. 4). From the point of view of the carrier transport and recombination, these results suggest that Y-doping can optimize the crystal quality of InSe and, in particular, the *c*-direction transport of intralayer carriers that enhanced the density and distribution of interlayer carriers, possibly due to the previously-confirmed (interlayer) stacking-faults elimination and the extremely-low carrier mass along the *c*-direction in InSe^34^. This subsequently results in the stronger interlayer exciton recombination (*P*_B_) in InSe:Y (Fig. 2c, d). Thus, we can infer that Y-doping-induced microstructure perturbations play a critical role in the *E*_*g*_ blueshift and carrier redistribution in vdW-layered InSe, which can also be confirmed by our first-principles calculations. As shown in our density function theory (DFT) calculation results (Fig. 2f–h and Supplementary Fig. 5), both the pristine and Y-doped InSe are indeed direct with the conduction and valence band maximums situating at the high-symmetric A point, which gives *E*_*g*_–values of 1.209 eV and 1.232 eV, respectively, totally consistent with the experimental data.

### Structural symmetry and PFM results

**Fig. 3:**
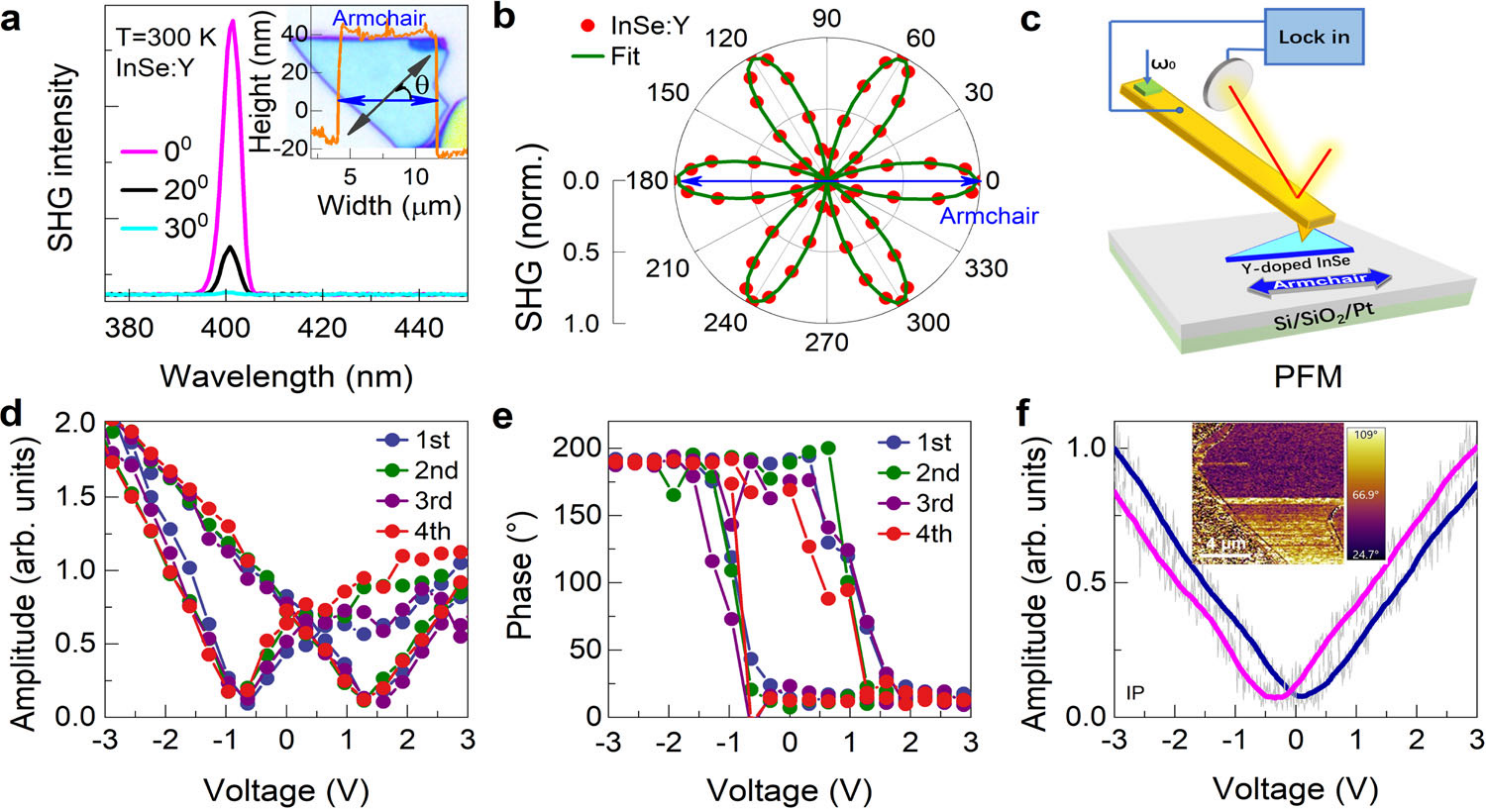
SHG and PFM of InSe:Y. **a** SHG intensity at angles of 0^0^, 20^0^, and 30^0^. The inset gives the exfoliated InSe:Y morphology with the thickness of ~50 nm measured by atomic force microscopy (AFM). **b** Polar plots of SHG intensities collected by rotating the polarizer as a function of the detection angle. **c** Schemes of the InSe:Y film structure and the setup for the PFM measurement. The local PFM amplitude (**d**) and phase (**e**) loops during the switching process of the InSe:Y flake with thickness of ~50 nm (results of four cycles). **f** IP PFM amplitude at low frequencies together with the IP phase image as inset.

2D γ-InSe belongs to the C^5^_3 v_ point group with noncentrosymmetry, possessing a strong second harmonic generation (SHG) effect^24^, and was theoretically predicted to exhibit sliding ferroelectricity^2^. Since we have optimized the crystal quality and the carrier transport, especially the intralayer carriers transport along OOP direction in γ-InSe by Y-doping, we further verify the symmetry and the piezo/ferroelectric properties in InSe:Y. A polarization-dependent SHG signal at 400 nm (an 800 nm laser source) is detected on a ~50 nm (about 20 unit cells) thick InSe:Y flake (inset in Fig. 3a) when rotating the angle (*θ*) of the sample (Fig. 3a), where *θ* = 0° means the incident polarized light is parallel to the AC direction of InSe. The *θ*-dependent SHG intensity (Fig. 3b) indicates an obvious 6-lobe structure, pointing to the *R3m* space group for InSe:Y, the same as the original InSe. In addition, the AC and ZZ directions can be easily distinguished, indicating the anisotropy of InSe (and InSe:Y) with broken inversion symmetry and directional selectivity. A similar SHG result can be observed in different InSe:Y samples (Supplementary Fig. 6), which should exclude the coincidence of the positive and negative charge centers that break the spatial inversion symmetry^1^. Next, piezoresponse force microscopy (PFM) is employed to explore both the piezoelectric and ferroelectric properties in InSe:Y. As schematically shown in Fig. 3c, a voltage-dependent PFM hysteresis loop is obtained at individual points on the same InSe:Y flake (inset in Fig. 3a) with the direct voltage swept from −3 V to +3 V and an alternating voltage of 0.5 V with a resonant frequency of ~350-kHz at the needle tip. Surprisingly, the vertical electric field applied between the probe and sample surface causes the ferroelectric-like amplitude response to show a butterfly loop with an opening of ~2 V, while the phase switches 180° at the same turning point (Fig. 3d, e). After cycling the test four times, the amplitude hysteresis loop remains in a butterfly shape and the phase transition remains at 180°. Additionally, the polarization switch on a clean surface is able to write reverse polarity domains at different voltages using the tip as the polarization electrode, further confirming the ferroelectricity in bulk-like InSe:Y (Supplementary Fig. 7). To exclude the random phenomena or experimental errors^35^, the OOP polarization is verified for InSe:Y flakes with different thicknesses (Supplementary Fig. 8 and Note 3) or at the low frequency (23-kHz) mode (Supplementary Fig. 9 and Note 3). Then, we determine the OOP effective-*d*_*33*_ piezoelectric constant (*d*) of uniform ∼7.5 pm/V for ∼50 nm thick InSe:Y flakes. Notice that with the thickness decrease from ~120 nm (about 48 unit cells) to ~14 nm (about 5 unit cells), the *d*-value experimentally demonstrates a monotonous increase from ~4.0 pm/V to 14.1 pm/V (Methods, Supplementary Note 3 and Table S1), suggesting that the thinner of the InSe:Y flakes, the stronger of the piezo/ferroelectricity. This *d*-value is remarkably larger than earlier reports including the new 3*R*-like MoS_2_/WS_2_ heterobilayers by a factor of ∼7^16^, the monolayer α-In_2_Se_3_ by ∼44 times^36^, and the bulk γ-InSe by ∼10 times^13,37^ (Methods, Supplementary Table 1), implying new piezo/ferroelectric enhancement mechanism in our InSe:Y material system. Simultaneously, at low frequency, the IP PFM (Fig. 3f) also shows a typical amplitude response as a butterfly loop with an opening voltage of ∼0.4 V and two distinct regions with an ~60° phase difference in the corresponding IP phase image (inset), demonstrating the simultaneous presence of IP ferroelectricity in InSe:Y. No similar phenomena can be observed in ordinary γ-InSe (Supplementary Fig. 10). Thereafter, the OOP and IP ferroelectricity raises an interesting possibility to trigger and enhance the spontaneous polarization of γ-InSe via Y-doping.

### Origin of FE in InSe:Y

**Fig. 4:**
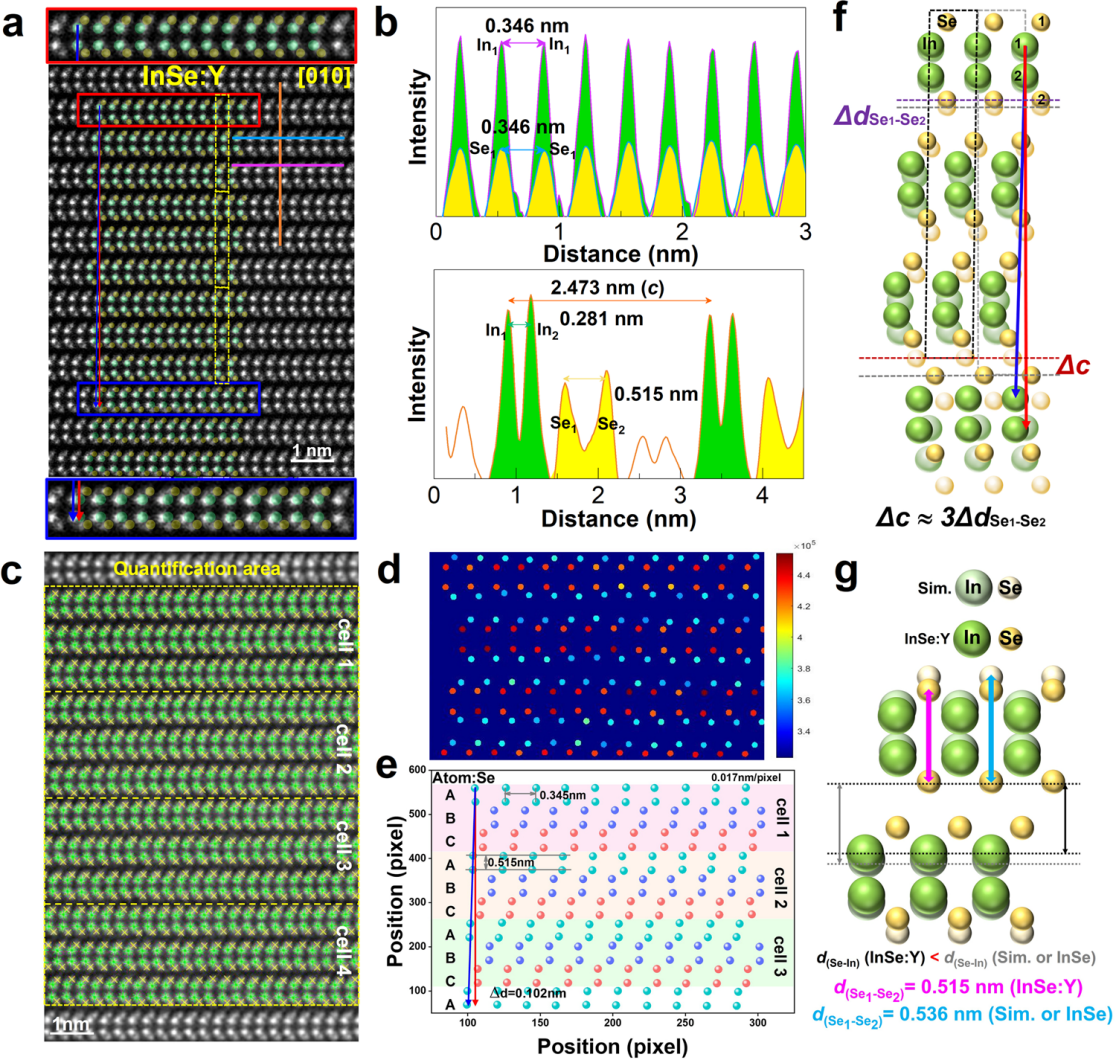
Microstructure analysis InSe:Y. **a** STEM-HAADF image of InSe:Y with magnified images at the top (bottom) for the red (blue) rectangles. The simulated γ-InSe atomic arrangements with green (yellow) dots representing In (Se) atoms are overlaid on the HAADF image, which demonstrates the continuous slip behavior layer-by-layer, as traced by the red (blue) arrows. **b** Intensity line profiles extracted from **a** with the upper along the horizontal purple (In_1_–In_1_) and sky-blue (Se_1_–Se_1_) lines and the lower along the vertical orange line. **c** Atomic positions of In (green stars) and Se (yellow crosses) identified by Calatom software. **d** Normalized intensities of the bright dots in **a**, with red (blue) points representing for In (Se). **e** The corresponding atomic positions of Se in **c**, showing the layer-by-layer sliding (Δ*d*) along the AC direction, well consistent with the results in **a**. **f** Schematic diagram of Y-doping-induced microstructural changes of the subtle rhombohedral distortion including intralayer compression along the *c*-axis (Δ*d*) and continuous interlayer pre-sliding (Δ*c*) in γ-InSe unit cell. **g** Schematic of Y-doping-induced compressions of Se_1_-Se_2_ spacing (*d*_(Se1-Se2)_) in Se-In-In-Se intralayer and Se-In dipole spacing (*d*_(Se-In)_) in vdW interlayer gap.

To get deep insight into the interplay of Y-doping-induced structural modifications and the intriguing ferroelectric property in InSe, we perform comprehensive scanning TEM-high angle annular dark field (STEM-HAADF) analyses to directly investigate the atomic configuration. As shown in STEM-HAADF images of InSe (Supplementary Fig. 11) and InSe:Y (Fig. 4a and Supplementary Fig. 12), both crystals indeed belong to the γ-phase with an ABC-stacking sequence (inset in Supplementary Fig. 11a). In sharp contrast, there is a high density of stacking-faults in InSe, but only a few in InSe:Y, in line with the previous TEM/SAED results. The top and bottom insets in Fig. 4a amplify the regions marked by red and blue rectangles, respectively, where the atomic arrangements of In and Se are overlaid on the HAADF image (green/yellow points for In/Se), demonstrating the doping-induced continuous sliding behavior along the AC direction, layer-by-layer with a relatively long periodicity (approximately 30 layers; Fig. 4a, e). Moreover, a localized inward compression of the lattice structure is also observed in InSe:Y. Indexing from the corresponding fast Fourier transform pattern (Supplementary Fig. 12b) of the HAADF image (Fig. 4a), the lattice distance of the (003) planes in InSe:Y is ~0.824 nm, i.e., smaller than that of the undoped (0.843 nm; Supplementary Fig. 11), or the simulated atomic structure (0.844 nm; Supplementary Fig. 1d). This gives a reduced lattice parameter *c* = 2.473 nm (~2.33%) for InSe:Y in contrast to the theoretical value (2.532 nm). Furthermore, the intensity line profiles (Fig. 4b) along the purple, sky-blue and orange lines in Fig. 4a show that the projected atomic distances of In_1_-In_2_, In_1_-In_1_, and Se_1_-Se_1_ (i.e., *d*_In1-In2_ = 0.281 nm, *d*_In1-In1_ = *d*_Se1-Se1_ = 0.346 nm) remain unchanged, but that of Se_1_-Se_2_ (*d*_Se1-Se2_ = 0.515 nm) is evidently smaller than the theoretical value (0.536 nm in Supplementary Fig. 1d). Detailed atomic arrangement analyses for different STEM samples combined with atomic quantification via Calatom^38^ based on custom MATLAB scripts also demonstrate the same results (Fig. 4c–e and Supplementary Fig. 14 and Note 4). It can then be concluded that Y plays a critical role in introducing anisotropic stress/strain along the *c*-axis in InSe during the crystal growth, which leads to a subtle rhombohedral distortion including intralayer compression along the *c*-axis and continuous interlayer pre-sliding at atomic scale (Fig. 4f) due to the low intralayer rigidity of In-Se bonding and the weak interlayer vdW-force. It is worth noting that the decrease in the intralayer Se-Se vertical distance shortens the bond length between anion (Se) and cation (In or trace-Y) atoms, definitely broadening the optical gap and increasing the binding energy in InSe:Y, which is consistent with the observed blueshift of *E*_*g*_ in optical characterization and is also proven by our first-principles calculations (Methods, Fig. 3f–h) and the X-ray photoelectron spectral results (Supplementary Fig. 15 and Note 5). More significantly, the offset of the atomic position of Se-Se along the *c*-direction and the interlayer pre-sliding along the AC direction with a long periodicity can induce the redistribution of charges, as discussed in the TR-PL results, and simultaneously decrease the vertical interlayer spacing between the bottom In atoms in the upper layer and the corresponding top Se atoms in the adjacent lower layer (Fig. 4g). This should enhance the interlayer net polarization in the vertical direction^14^ and also introduce an additional polarization vector along the AC direction to strengthen the IP polarization, resulting in robust OOP and IP ferroelectricity, as observed by PFM.

It has been theoretically predicted that a small twist or strain difference can give rise to Moiré pattern in the graphitic bilayer of BN, AlN, ZnO, MoS_2_, GaSe, etc, or in bulk vdW InSe with *R3m* symmetry^14^ due to the relatively low sliding potential. And certain stacking in those bilayers that breaks the symmetry and makes two layers unequivalent may lead to net polarizations. For instance, Fei et al.^39^ reported an interlayer sliding potential (~meV) several orders of magnitude lower in WTe_2_ than that of conventional ferroelectrics. Accordingly, interlayer sliding ferroelectricity was experimentally demonstrated in BN^9,15^, graphene^10^ and TMDs^16^. Undoubtedly, in the case of InSe:Y, the sliding potential along the continuous interlayer pre-sliding direction should further decrease. It can then be inferred that, although ordinary γ-InSe theoretically possesses spontaneous polarization that can be reversed by external electric fields, and Wu et al.^13^ even predicted a weak OOP polarization of ~0.08 µC/cm^2^ for vdW bulk γ-InSe, the abundant naturally-existing planar stacking-faults weakened the probability for an experimental observation of the really weak intrinsic ferroelectricity. In contrast, our structure control approach fantastically stabilizes a subtle distorted rhombohedral configuration with strengthened OOP and IP spontaneous polarizations, thus arousing the robust OOP and IP ferroelectricity in InSe:Y. Our observations provide a strategy to tune and optimize the piezo/ferroelectric property in 2D vdW-layered semiconductors.

## Discussion

So far with the help of trace Y dopant, we realized unconventionally robust OOP and IP ferroelectricity in the high crystal quality vdW-layered γ-InSe semiconductor due to the natural stacking-faults elimination and a subtle distorted rhombohedral configuration, underlining the striking role of doping-induced stress/strain in the manipulation of the fundamental properties of condensed matter. This investigation opens up a pathway to tuning the ferroelectric properties for expanding the member of 2D vdW-layered ferroelectric semiconductors and also gives new insight into the interplay of the microstructure and physical properties in 2D layered semiconductors. The high and uniform effective piezoelectric constant covering a wide thickness range suggests the promising application in ferroelectric information storage/processing and self-driving high-speed photonic devices made of InSe narrow-gap semiconductor with a thickness from bilayer to bulk-like states.

## Methods

### Sample growth and transfer

Samples involved in this work are pure InSe and InSe:Y. The growth environment and process of both single crystals are the same with the Bridgman method^26,28^. The small particles of high-purity In (99.999%) and Se (99.999%) are mixed in a closed quartz ampoule with a non-stoichiometric ratio In:Se = 0.52:0.48, and the vacuum degree is 10^−3 ^Torr. The material is melted in the high temperature zone (~700 °C), single crystals are grown in the temperature gradient zone (10–15 °C/cm), and crystal annealing is mainly carried out in the low temperature zone (300–550 °C). For the preparation of InSe:Y, 1 at% Y is mixed with In to form In:Y alloy first. The rest of the synthesis steps is the same.

InSe and InSe:Y flakes for SHG and PFM analyses are prepared by employing a scotch-tape mechanical exfoliation method. The thickness and surface morphology of the flakes are characterized by AFM (Multimode 8, Bruker, USA).

### Structural characterization

The morphology and composition measurements are performed on the Zeiss Gemini 450 equipped with an EDS (Ultim Extreme, Oxford, UK). XRD patterns are obtained on the Bruker D8 Discover (Germany) with a monochromatic Cu *Kα* radiation source (*λ* = 0.15406 nm). The X-ray profiles are recorded in *θ*–2*θ* scan mode with 2*θ*∈(10°, 80°). Cross-section TEM specimens are prepared by dual-beam FIB system (Helios G4 UX, FEI, USA) using Ga-ion accelerating voltage ranging from 2 to 30 kV, followed by ion-milling (Gatan 691, Gatan, USA) operated at 2 kV to remove the residual amorphous layer. TEM images and EDS analysis are acquired at 200 kV using a JEM-2100F JEOL equipped with an Oxford EDS detector (X-Max 80 T, Oxford, UK). Atomic-resolution imaging measurements are performed on an aberration-corrected HAADF STEM (AC-HAADF-STEM) (JEOL Grand ARM300, Japan) equipped with JEOL dual 158 mm^2^ SDD detectors for atomic level EDS analysis. To improve the signal-to-noise ratio and minimize the drift or distortion of AC-HAADF-STEM images, six serial frames are acquired with a short dwell time (2 μs·pixel^−1^). The image series are aligned and superimposed.

### Chemical composition analysis

TOF-SIMS analysis is carried out on ION-TOF GmbH TOF SIMS 5 (Germany) with detection limit at *ppm* ~ *ppb* level, lateral resolution <70 nm, depth resolution <1 nm. The AXIS Ultra DLD (Shimadzu Kratos, Japan) multifunctional X-ray photoelectron spectroscopy provides the chemical information of InSe:Y.

### PFM measurement

PFM measurements at the dual amplitude resonance tracking (DART) mode are performed on a commercial scanning probe microscopy (SPM) system (Asylum Research Cypher, Oxford, UK) with conductive Pt-coated silicon cantilevers. A soft tip with a spring constant of 2.8 N m^−1^ was driven with an AC voltage (*V*_*ac*_ = 0.5 V) under the tip-sample contact resonant frequency (~350 kHz). PFM analysis at low frequency under non-resonant mode for the same typical flakes were carried out on Multimode 8 AFM (Bruker, USA).

The LithoPFM mode of the Asylum Research software is used for permanent domain writing of InSe:Y flake’s surface, where the PFM tip carries reverse DC biases (*V*_*dc*_ = ±5 V) when moving in contact mode across the surface.

### Derivation of the effective piezoelectric constant *d*

To quantitatively obtain the piezoelectric information, the contact resonance of the probe can be reduced to a Simple Harmonic Oscillator (SHO) model using IGOR Pro (Asylum Research). The inverse of the optical lever sensitivity (InvOLS) of the probe is calibrated before the experiment to convert the voltage to displacement. During PFM measurements, the experimental parameters used for InSe:Y were *V*_*ac*_ = 0.5 V, and the range interval of *V*_*dc*_ is continuously increased from –3~3 V to –7~7 V. The effective piezoelectric constant *d* in the weak-indentation limit satisfies the relation with *d*_*33*_, *d* = 0.5*d*_*33*_.

In PFM experiments, the amplitude of the probe can be expressed as below if the sample is polarized in the vertical direction,

1 $${A}_{{tip}}={A}_{s}\cdot C={d}_{33}^{{eff}}\cdot {V}_{{ac}}\cdot C$$

where *A*_*tip*_ is the vertical amplitude of the probe, *A*_*s*_ is the vertical amplitude of the sample surface due to the piezoelectric effect, *V*_*ac*_ is the amplitude of the AC voltage applied on the probe (Drive Amplitude), *C* is a factor related to the drive frequency, and ${d}_{33}^{{eff}}$ is the effective piezoelectric coefficient related to the polarization state of the material.

The spatially-resolved piezoelectric coefficient ${d}_{33}^{{eff}}$ is found to be stable with a uniform value of ~14.1 pm/V for the thickness of 14 nm (~5 unit cells) InSe:Y, and slightly decreased to ~4.04 pm/V as the thickness increases to 120 nm (~48 unit cells); see Supplementary Table S1. Notice that the flakes are easy to be destroyed under the role of voltage when the thickness is further decreased, possibly due to the voltage-induced thermal effect.

### Optical characterization

Raman spectra are recorded on a Renishaw inVia confocal Raman microscope equipped an excitation wavelength of 532 nm and a ×100 objective (*NA* = 0.85), which provides a spectral resolution of higher than 0.5 cm^−1^ and a spatial resolution of less than 0.5 μm. Parallel and perpendicular polarization geometries are configured, and the polarization angle can be controlled continuously by rotating either the polarizer or the sample. The laser intensity is set at ~1 mW/cm^2^ order of magnitude to avoid the excitation-induced thermal effect.

SHG measurements are performed at room temperature using a confocal microscope setup in reflection geometry. A mode locked Ti: sapphire laser (Tsunami) at 800 nm (pulse width 100 fs, repetition frequency 80 MHz) is used as the excitation source. The collimated laser beam is passed through a Glan Thompson polarizer (GTH10M-AM-A, Thorlabs) followed by a wave plate (AQWP05M-600, Thorlabs), which is focused by a ×50 objective lens (Zeiss, 0.75 NA) onto the sample after passing through a beam splitter (CCM1-BS013/M, Thorlabs). The SHG signal is collected by the same objective and directed through a dichroic beam splitter to a spectrometer equipped with a 300 grooves/mm grating and a nitrogen-cooled silicon charge-coupled device.

A Fourier transform infrared spectrometer (Vertex 80 v, Bruker) covering a wavelength range of ~0.4 − 15 µm is employed for optical transmission and reflectance measurements. TR-PL measurements are implemented with a reflectance geometric configuration. The Tsunami Ti: sapphire laser described in SHG measurements is used as the excitation source. The signal is recorded by a Hamamatsu synchroscan streak camera with an S1 photocathode (temporal resolution better than 10 ps). Low pulsed excitation densities of ∼μJ/cm^−2^ are ensured to avoid the excitation-induced thermal or extrinsic effects. For temperature-dependent measurements, the sample is mounted on the cold head of a circling cryogenerator, which allows for the temperature changing from 4 K to ambient temperature.

### DFT calculations

The first-principles calculations of structural optimization and electronic structure are performed using the DFT as implemented in the Vienna ab initio simulation package (VASP) code. The projector augmented-wave (PAW) pseudopotentials with an energy cutoff of 450 eV for the planewave basis set are employed. For the structural optimization, the Perdew–Burkes–Ernzerhof (PBE) form of the generalized gradient approximation (GGA) to the exchange-correlation functional is used. All lattice vectors and atomic positions are relaxed until the total force on each atom is lower than 0.01 eV Å^−1^. The electronic structures are determined using the HSE06 hybrid exchange-correlation functional so that the *E*_*g*_ matches the experimental value. A 12-atom primitive cell together with 7 × 7 × 1 k-point mesh is employed for band structure of the pure crystal, whereas a 144-atom supercell and 3 × 3 × 1 k-point mesh were used for the Y-doped system. Using HSE06, γ-InSe is determined to have a 1.209 eV direct *E*_*g*_ located at the high symmetric A point (Fig. 2f). To study the effect of Y-doping, we construct a supercell containing 144 atoms, and replace one In atom with Y. The ratio is 1/144, which is close to the experimental doping concentration. We comparatively calculate the electronic band structures of the Y-doped and pristine supercell. Due to the large computational cost, we only calculate the band along Γ-A-H (Fig. 2g, h). While keeping the direct *E*_*g*_ at A, the Y-doping makes the *E*_*g*_ blueshift to 1.232 eV.

## Supplementary information

Supplementary Information

## Data Availability

The data supporting the findings of this work are included within the paper and its Supplementary Information files. Extra data are available from the corresponding authors upon reasonable request.

## Code availability

The codes used to process and analyze the data presented in this work are available from the corresponding author upon request.
