# Supplementary material for: Sliding ferroelectricity in van der Waals layered γ-InSe semiconductor
Source: Nat Commun. 2023 Jan 3;14:36. doi: 10.1038/s41467-022-35490-0 (PMC9810696; doi:10.1038/s41467-022-35490-0)
Supplement: Supplementary file 1 — Supplementary Information [file 41467_2022_35490_MOESM1_ESM.pdf]

# Supplementary Information

## Sliding ferroelectricity in van der Waals layered γ-InSe semiconductor

Fengrui Sui[1], Min Jin[2], Yuanyuan Zhang[1], Ruijuan Qi[1,3]*, Yu-Ning Wu[1], Rong Huang[1], Fangyu Yue[1]* & Junhao Chu[1]

[1]Key Laboratory of Polar Materials and Devices (MOE), School of Physics and Electronic Science, East China Normal University, Shanghai 200062, China.

[2]College of Materials, Shanghai Dianji University, Shanghai 201306, China.

[3]State Key Laboratory of Functional Materials for Informatics, Shanghai Institute of Microsystem and Information Technology, Chinese Academy of Sciences, Shanghai 200050, China.

*Correspondence to: rjqi@ee.ecnu.edu.cn; fyyue@ee.ecnu.edu.cn

**This PDF file includes:**

Supplementary

Notes 1 - 7

Figs. 1 - 15

Table 1

References (1 - 30)

## SUPPLEMENTARY NOTE 1: PHASE STRUCTURE OF INSE

Monolayer InSe crystal structure consists of covalently bonded atoms (Se-In-In-Se), and the layers are held together by weak vdW-forces[1]. This special structure makes it easier for exfoliating. The structure determines the materials' properties, and therefore the phase identification of 2D InSe is of utmost importance. As shown in Fig. S1a-c, 2D InSe possesses three different phase structures (β, ε, and γ) with different arrangements of atoms and stacking layers. The β- and ε-phases have the same lattice parameters: *a*=*b*=4.05 Å, *c*=16.93 Å, but belong to different space groups. The β-phase with hexagonal lattice structure belongs to $D_{6h}^{4}$ (*P6₃/mmc*) centrosymmetric space group, with A$\bar{A}$-style stacking[2]. Interestingly, ferroelectricity in centrosymmetric β-InSe has been reported[3,4]. Further studies would be helpful to explore its origin through comprehensive structural analyses. The ε-phase with hexagonal lattice structure belongs to $D_{3h}^{1}$ ($P\bar{6}m2$) non-centrosymmetric space group, with AB-style stacking[5]. The γ-phase, possessing rhombohedral lattice structure with lattice parameters of *a*=*b*=4.00 Å and *c*=25.32 Å, belongs to $C_{3v}^{5}$ (*R3m*) noncentrosymmetric space group with ABC-style stacking[6], where the In atoms in one layer are aligned with the Se atoms in the other, breaking down the mirror-plane symmetry that is characteristic of monolayer InSe. The broken mirror symmetry causes interlayer charge transfer through hybridization between the occupied states of one layer and the unoccupied states of the other layer, generating an out-of-plane (OOP) electric dipole moment[7] that may lead to piezoelectric or ferroelectric behaviors.

Commonly, InSe crystal grown by the Bridgman method possesses γ-phase with plenty of stacking-faults. As reported in our previous report[8], it is difficult to distinguish the actual polytype of InSe through XRD and Raman, so we performed detailed selected area electron diffraction (SAED) analysis by comparing with the simulated one (Fig. S1e) to confirm the γ-phase of InSe:Y in this work. Moreover, the existence of stacking-faults in the undoped InSe can be clearly seen from the TEM image (Fig. S2c) and the obvious diffraction "straight line" appeared in the SEAD pattern of InSe[9], but no same phenomenon can be seen in InSe:Y (Fig. S2d), indicating more stable

layer-to-layer alignment in the Y-doped InSe. Further STEM-HAADF images clearly show both InSe and InSe:Y exhibit γ-phase like ABC-stacking style with many stacking-faults in InSe but only a few in InSe:Y.

## SUPPLEMENTARY NOTE 2: BAND STRUCTURE ANALYSIS

According to the Tauc-plot, the optical bandgap ($E_g$) of semiconductors can be obtained from transmission spectra[10,11]. The formula is presented as,

$$\alpha h\nu=\beta(h\nu-E_g)^n \quad (1)$$

Where $\alpha$, $h\nu$, and $\beta$ represent absorption coefficient, photon energy, and band-tail parameter, respectively. For 2D InSe flakes, the parameter $n$ equals to 1/2, i.e., for a direct semiconductor[12], which can be supported from the smooth and steep absorption edge (Fig. 2a) and the TR-PL results (Fig. 2c,d). The inset in Fig. 3a for the Tauc-plot shows $E_g$=1204 meV for InSe and 1229 meV for InSe:Y with a difference of ~25 meV, consistent with the results of reflectance spectra (Fig. 2b) and also the report[13]. Notice that the reflectivity of both crystals is extremely low due to the high absorption coefficient of InSe flakes, but the blue-shifted reflectance edge can be well distinguished although the blue-shift value is only ~24 meV.

TR-PL images at ~0 ps of both crystals show a double-peak structure, a low energy peak $P_A$~1240 eV and a high energy one $P_B$~1300 meV (Figs. 2c and S4), of which $P_A$ is ascribed to the $E_g$-related bandedge transition by considering that i) no lower energy peak but $P_A$ can be observed within a wide range variation of excitation density; and ii) the peak energy of $P_A$, which has a slightly longer decay time (2.17 – 2.37 ns) than that of $P_B$ (1.22 – 1.56 ns), is consistent with that of absorption spectra. That is to say, $P_A$ should be related to the intralayer $E_g$-transition of InSe. This ~2 ns lifetime is also consistent with the direct transition in *R3m*-type samples[14]. However, the natural stacking-faults in InSe can evidently influence the lifetime of $E_g$-related non-equilibrium carriers, but in InSe:Y the lifetime is almost a constant due to the doping-induced elimination of stacking-faults. $P_B$, with a blue-shift of only ~60 meV compared to $P_A$, has a similar time constant of $P_A$, suggesting that $P_B$ is neither from the other indirect valleys nor the higher

excited states within the intralayer, but can be ascribed to the interlayer transition due to the interlayer pre-sliding in γ-InSe (both the interlayer sliding in InSe:Y and the stacking-faults in InSe can introduce the interlayer transition, as widely reported in 2D interlayer sliding and/or Moiré heterostructures by PL or TR-PL[7,14,15]). Notice that Y-doping can optimize the rise of the non-equilibrium carriers, and strengthen the high energy peak $P_B$. More importantly, the Y-doped InSe has a relatively homogeneous TR-PL image shapes at different spatial positions (Fig. S4b, e, f). Instead, the pristine InSe shows evident nonuniformity at different positions absolutely due to the natural stacking-faults (Fig. S4a, d). From the point of view of carrier transport, Y-doping also enhances the crystal quality by brightening the interlayer emission, providing uniformly derivate parameters.

## SUPPLEMENTARY NOTE 3: VERIFYING FERROELECTRICITY IN INSE:Y BY PFM

PFM is a powerful technique for the characterization of ferroelectric materials by measuring the dynamic electromechanical response[16]. By definition, a ferroelectric material should possess spontaneous polarization that is switchable, showing local PFM amplitude and $180^0$ phase loops. As reported, the application of bias can lead to a wide variety behavior during PFM measurements including Joule heating, charge injection, and ionic motion[17-19], and several strategies can be used to explore the intrinsic ferroelectric behavior through varying the $V_{ac}$ amplitude, frequency and tip-sample contact stiffness, etc[8,18,20].

In this work, dual amplitude resonance tracking (DART)-PFM mode on a commercial scanning probe microscopy (SPM) system (Asylum Research Cypher, Oxford, UK) was used to ensure strong signal-to-noise ratio[21]. We carried out local switching tests for both InSe:Y and InSe flakes under the same conditions by applying a bias between the PFM cantilever and the sample on the Pt coating Si substrate in the off-field state to restrain the contributions of electrostatic force to the signal. A soft tip with a spring constant of 2.8 N $m^{-1}$ was driven with an AC voltage ($V_{ac}$=0.5 V) under the tip-sample contact resonant frequency (~350-kHz). As demonstrated in Fig. S8, robust OOP ferroelectric polarization can be detected in all InSe:Y flakes but not for InSe flakes

(Fig. S10). We have confirmed the ferroelectric behavior in InSe:Y by varying $V_{ac}$ amplitudes, which is a necessary step to establish the veracity of PFM hysteresis measurements. Further, domain writing at the LithoPFM mode of the Asylum Research software with reverse DC biases ($V_{dc}$=±5V) in Fig. S7 also proofs the ferroelectricity in InSe:Y. The experiment demonstrates evident thickness-dependent ferroelectric polarization when the unit cell value of InSe is changed from ~5 to 48 (Table S1).

Moreover, as the in-plane (IP) piezoresponse is orientation-dependent and proportional to the IP polarization component, perpendicular to the cantilever of the AFM tip, it is hard to characterize the IP ferroelectric in γ-InSe:Y owning three AC directions. In this case, to verify the OOP and IP polarizations in InSe:Y, concerted vertical (OOP) and lateral (IP) PFM are carried out under off-resonance mode with low frequency (23-kHz) on Multimode 8 AFM (Bruker, USA), which is conducive to obtain the intrinsic piezoresponse signals from samples by reducing the interference from the tip. As shown in Fig. S9, butterfly loops of the PFM amplitude signals and the distinct 180° switching of the phase signals in both OOP and IP directions can be obtained with four times cycles, corroborating the robust OOP and IP ferroelectric polarizations in InSe:Y flakes.

## SUPPLEMENTARY NOTE 4: COMPREHENSIVE STEM-HAADF IMAGES ANALYSES

Due to the incoherent scattering intrinsic, the contrast of HAADF imaging is proportional to $Z^{1.7}$ [(22)]. As the atomic number of In (49) is bigger than that of Se (34), the bigger (smaller) bright dots represent In (Se) atom columns in STEM-HAADF images. To eliminate the possible errors during measurements, we perform comprehensive analyses on the STEM-HAADF images and the corresponding FFT patterns for four different samples prepared by FIB at random positions on InSe:Y crystal; see Figs. 4a and S14, where the yellow dotted rectangles mark the *γ*-InSe unit cells and the blue arrows comparing with the vertical red arrows trace the minute sliding by layers. Through the detailed atomic distance (both vertical and horizontal) measurements for In-In, Se-Se and vdW gap in the HAADF images, we observe the similar microstructure changes for the four

InSe:Y STEM samples, including atomic displacements of Se along *c*-direction, Se-In-In-Se intralayer compression and continuous layer sliding along the AC direction with a relative long periodicity (about 30 layers).

To confirm the intrinsic layer-by-layer slip but not from the drift during the image acquiring, we obtained HAADF images of the same interested area with different scan rotations including $0^o$, $90^o$ and $180^o$. As shown in Fig. S13, layer-by-layer slip can be observed with scan rotations of $0^o$ and $180^o$, demonstrating the same pre-sliding features; however, no atomic sliding or drift can be observed with the scan rotation of $90^o$.

## SUPPLEMENTARY NOTE 5: XPS ANALYSES

XPS surface analysis is used to measure the Fermi levels in InSe:Y. As shown in Fig. S15, blue shifts (increase in energy) are observed in the binding energy of In and Se. For InSe:Y, the binding energy of Se *3d* shifts from 54.5 eV to 54.8 eV with an increment of 0.3 eV, and simultaneously the peaks of In $3d_{5/2}$ and In $3d_{3/2}$ shift from 444.5 eV to 445.1 eV and 452.2 eV to 452.8 eV with increments of 0.6 eV. The binding energy of XPS spectra is referenced to Fermi level in the material, so the blue-shifts of binding energy can be interpreted by the moving up of the Fermi level in the semiconductor[23], i.e., the strengthened binding energy in InSe:Y.

## SUPPLEMENTARY NOTE 6: CONSIDERATION OF RARE-EARTH Y-DOPING

Elemental doping is a commonly used high-entropy strategy for tuning the material property and electronic structure, which results in many novel physical/chemical performances[24,25]. Due to their unique optical, magnetic, and electrical properties, rare-earth ions have been extensively explored as active dopants to invoke rich optical, electrical, magnetic, and catalytic properties by altering the crystallographic phase, morphology, and size. Moreover, the beauty of using rare-earths is that even in small quantities, they can significantly alter material properties. It suggests that rare earth elemental doping strategy is a useful and feasible structural controlling approach to manipulating the fundamental properties of 2D vdW-layered materials.

We have tried different elemental dopants for InSe, such as Bi, Y and Dy. We find that different elemental dopants play striking but different impacts on the appearance and microstructure (the interlayer or the intralayer atomic configurations) of InSe crystals. For instance, the 0.01at% Bi-doping in InSe leads to much more stacking-faults and a shrinkage in the in-plane lattice parameters, but almost did not change the interlayer/intralayer atomic distances, which may be related to the doping-induced strain only along the in-plane direction. Furthermore, the Bi-doping induced in-plane strain has little impact on the appearance of InSe, while interestingly the doping of Y and Dy obviously changes the intrinsic plasticity of InSe. Further HAADF-STEM investigation demonstrates that the rare-earth (Y and Dy) doping in InSe may introduce out-of-plane strain in InSe during the crystal growth, resulting in the fantastic microstructure modifications including the stacking-faults elimination and a subtle rhombohedral distortion due to the intralayer compression and continuous interlayer pre-sliding. We strongly believe that they may also lead to other novel mechanical, optical, and electrical properties.

## SUPPLEMENTARY NOTE 7: OUTLOOK FOR APPLICATIONS

Considering the facts that i) the suitable optical bandgap of InSe:Y that has been determined as ~1.2 eV by experiment and calculation, ii) the ultrafast recombination process (in sub-nanosecond orders of magnitude), and iii) the strong OOP and IP ferroelectric polarization, especially the IP spontaneous ferroelectric polarization along the AC direction, it is promising for applications in self-driving planar high-speed (~GHz) infrared detectors or ferroelectric photovoltaics, and data storage devices if the sliding dynamics for the polarization inversion can be monitored and controlled, e.g., by the in-situ biasing STEM technique to visualize the adjacent interlayer sliding.

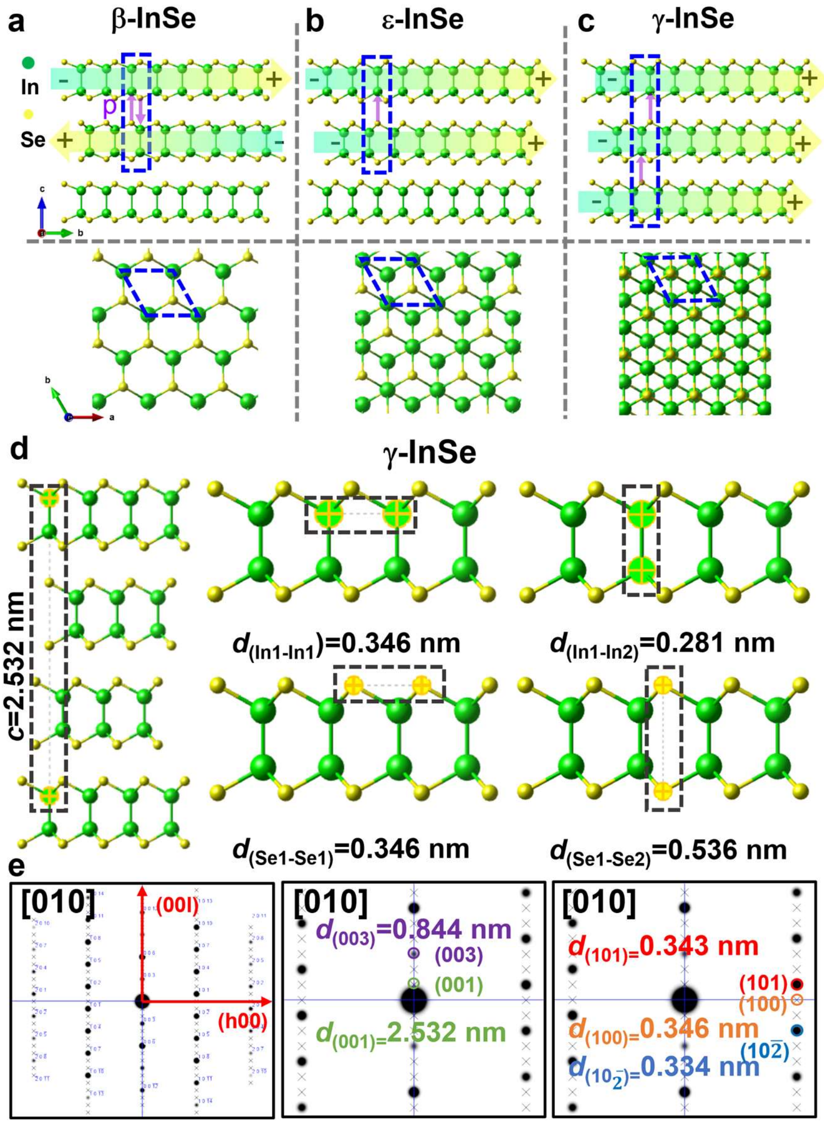


**Figure S1. The simulated atomic structure and SAED pattern. a**, **b**, **c**, Structural schemes: side-view (**upper**) and top-view (**lower**) of β-InSe (**a**), ε-InSe (**b**) and γ-InSe (**c**), where ε and γ-InSe exhibit the IP (horizontal along the AC direction; rightward arrows) and OOP (vertical to interlayer; upward arrows) polarizations. **d**, The atomic structure of γ-InSe projected along the [010] zone axis using *R3m* space group, showing the theoretical parameter of *c* and the projected distances of $In_1$-$In_2$, $Se_1$-$Se_2$, $In_1$-$In_1$ and $Se_1$-$Se_1$. **e**, SAED pattern of γ-InSe projected along the [010] zone axis, demonstrating the geometry of the main diffraction points and the corresponding lattice planes distances.

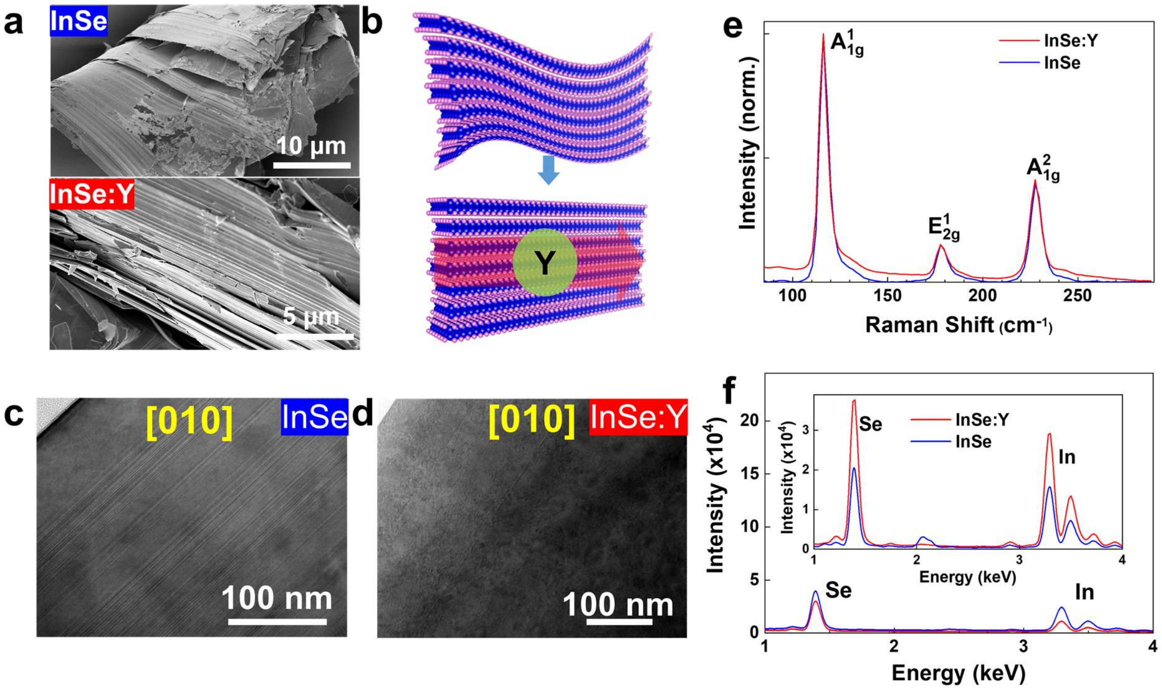


**Figure S2. Structural and chemical composition characterization**. **a**, SEM images of InSe and InSe:Y. **b**, Schematic illustration of the plasticity of InSe (**upper**) and the influence of Y-doping (**lower**). **c**, Low magnification TEM image of InSe. **d**, Low magnification TEM image of InSe:Y. **e**, Raman spectra of InSe and InSe:Y. **f**, SEM and TEM-EDS spectra of InSe and InSe:Y.

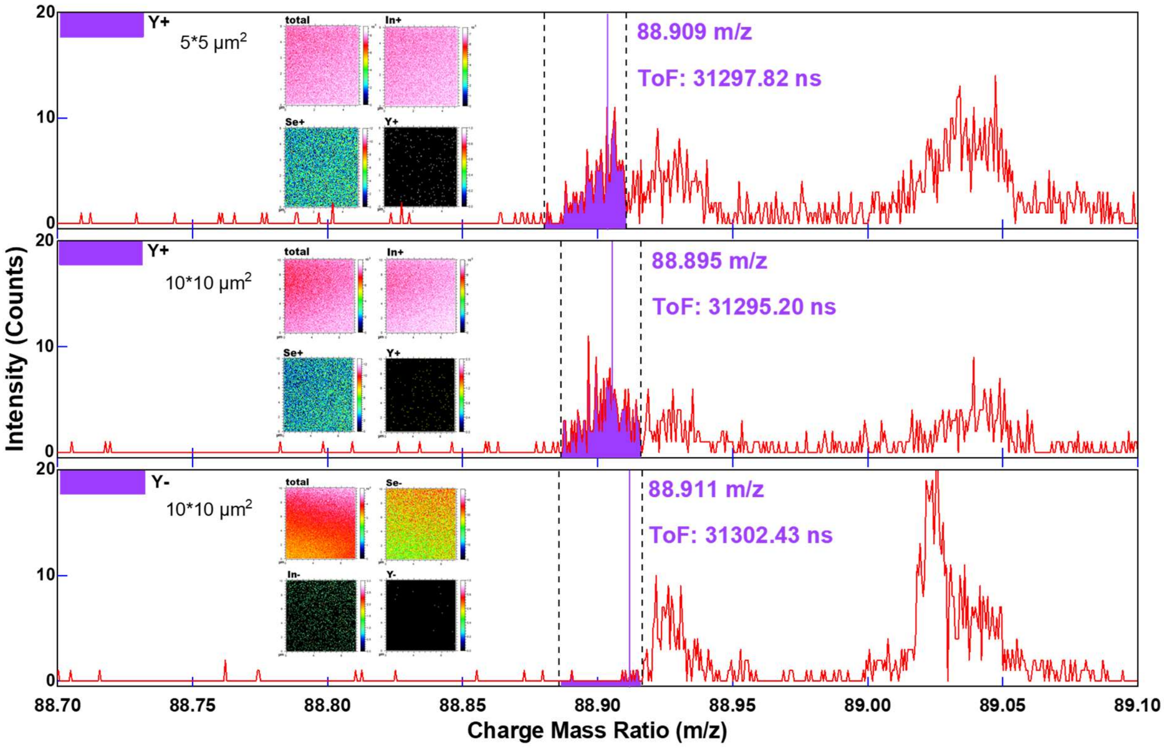


**Figure S3.** TOF-SIMS results of InSe:Y obtained at areas of 5x5 μm$^2$ a and 10x10 μm$^2$ b at the positive ion mode, showing an obvious distribution of positive ions of In and Y. The results obtained at the negative ion mode demonstrate an obvious distribution of negative ions of Se but without negative Y, indicating the positive valence state of Y in InSe.

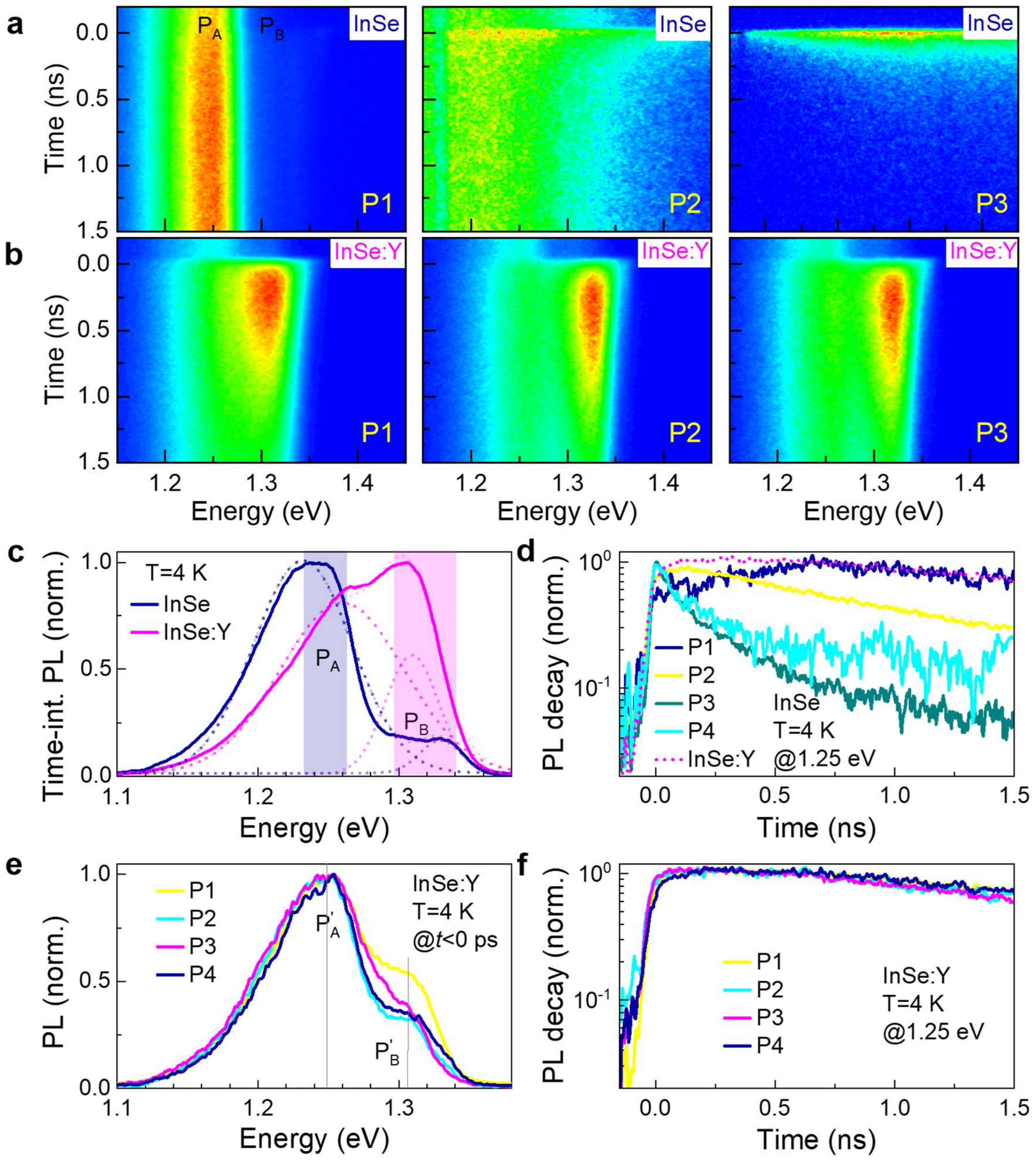


**Figure S4. TR-PL results of InSe and InSe:Y at 4 K. a**, **b**, TR-PL images at different positions on InSe (**a**) and InSe:Y (**b**). **c**, Time-integrated PL spectra of InSe and InSe:Y, together with the Gaussian fit results. **d**, PL decay curves extracted from TR-PL images at different positions of InSe. The curve of InSe:Y is also shown to make a comparison. **e**, Transient PL spectra of *t*<0 ps at different positions on InSe:Y. **f**, PL decay curves extracted from TR-PL images at different positions on InSe:Y.

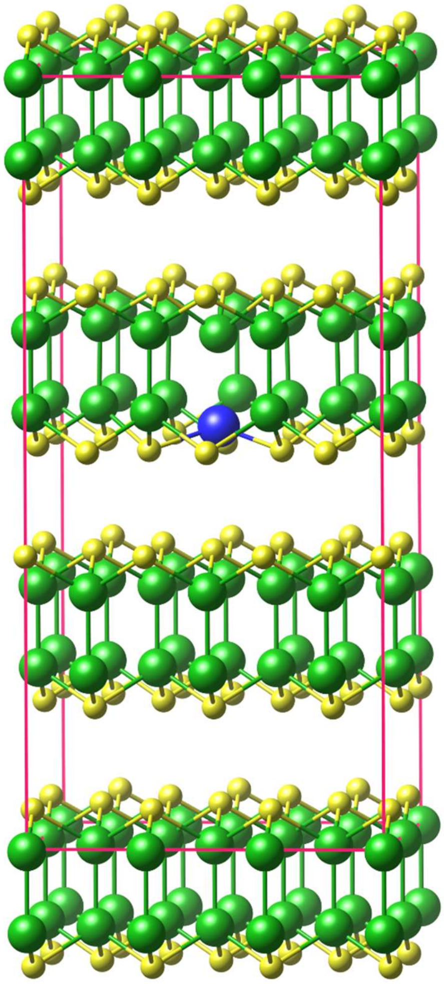

**Figure S5. The supercell structure used in our DFT calculations.** Blue – the Y atom, green – In atoms, and yellow – Se atoms.

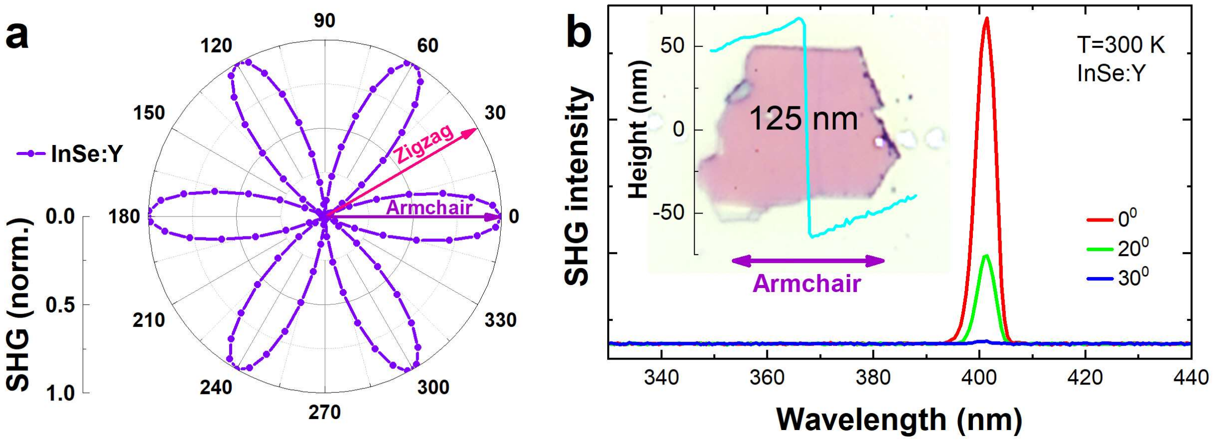


**Figure S6. Additional SHG. a**, Polar plots of SHG intensity collected by rotating the polarizer as a function of the detection angle (**left**). **b**, SHG intensity at angles of $0^0$, $20^0$, and $30^0$ (**right**). Inset gives the morphology of exfoliated InSe:Y flake with the thickness measured by AFM.

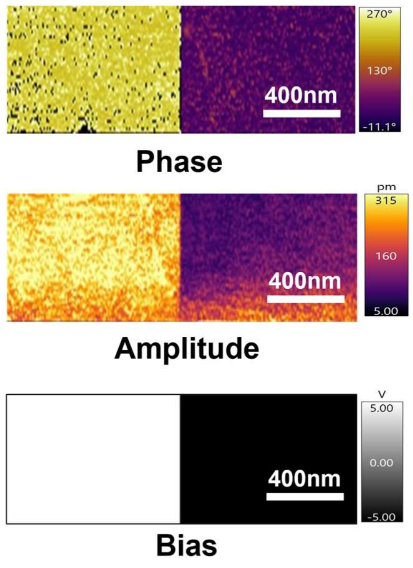


**Figure S7. Ferroelectric polarization switching by PFM for InSe:Y flake.** Domains are written onto the InSe:Y flake in Fig. **3** with reverse DC biases of –5 V for the left rectangle and 5 V for the right rectangle. The PFM phase and amplitude maps show the corresponding poled areas.

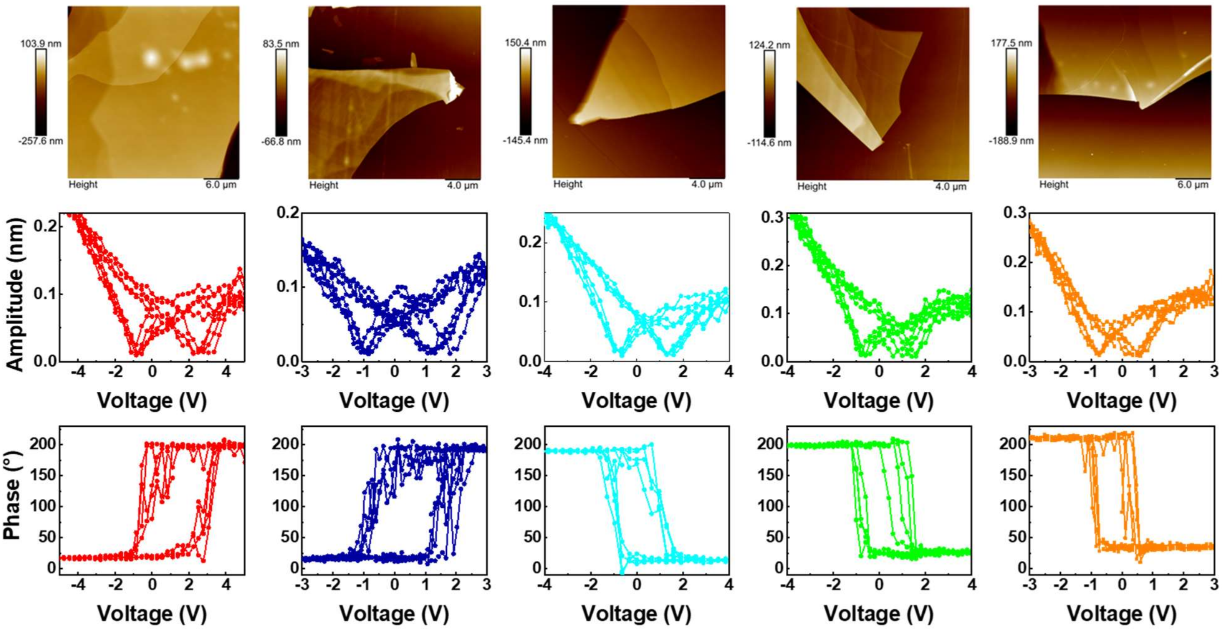


**Figure S8. Additional PFM results at high frequency.** The local PFM amplitude and phase loops (results of four cycles) during the switching process of InSe:Y flakes with different thicknesses (~50 – 170 nm measured by AFM) under the resonant mode (350-kHz).

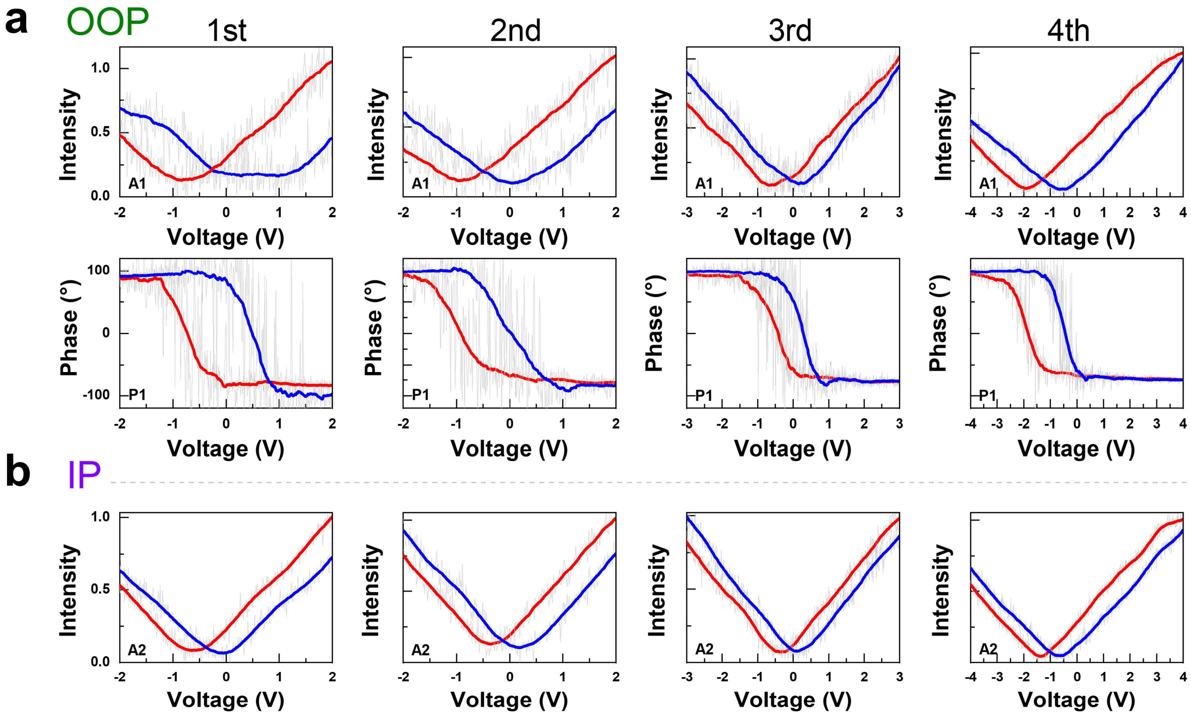


**Figure S9. Additional PFM results at low frequency. a**, **b** The local OOP PFM amplitude and phase loops (**a**) and IP PFM amplitude (**b**) of the same InSe:Y flake in Fig. **3** obtained synchronously at low frequency under the non-resonant mode (23-kHz, four cycles).

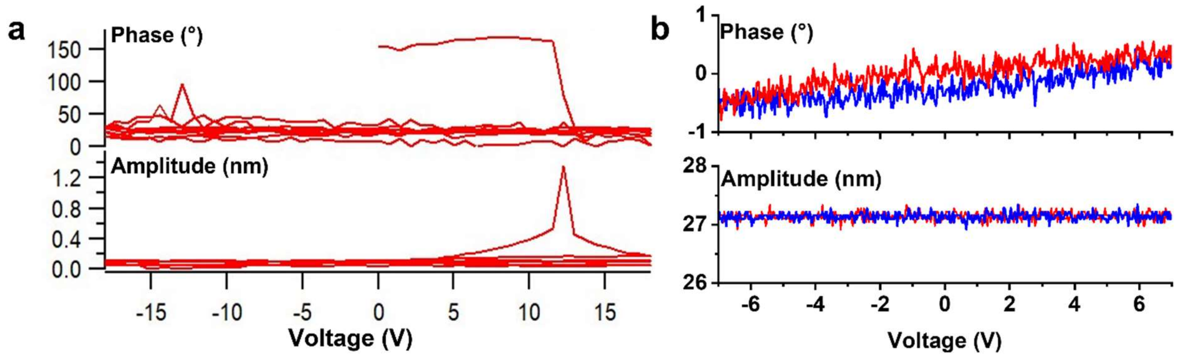


**Figure S10. PFM results of InSe flakes.** The OOP local PFM amplitude and phase loops (**a**) and the IP local PFM amplitude and phase loops (**b**) of InSe flakes obtained under the same condition as that of InSe:Y flakes, which show no ferroelectric-related PFM properties.

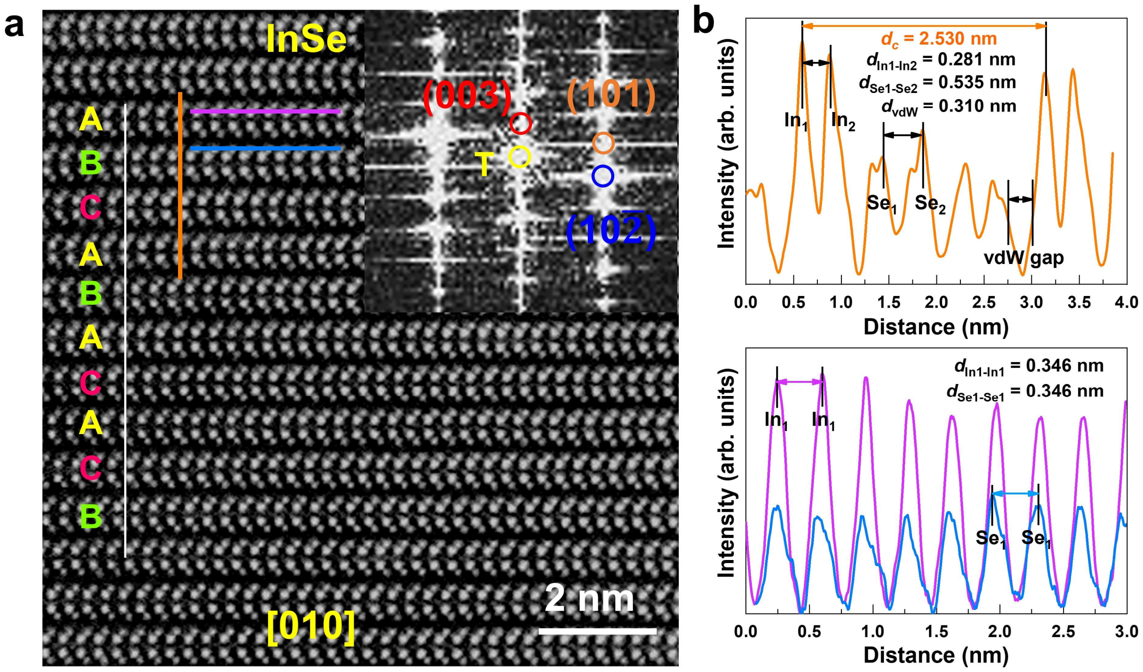


**Figure S11. Microstructure analysis of InSe. a**, HAADF image projected along the [010] zone axis showing the ABC γ-phase like stacking style along the white line with many stacking-faults, in line with the TEM results in Fig. **1d** and Fig. **S2c**. The inset is the corresponding FFT pattern. **b**, Intensity line profiles along the purple, sky-blue and orange lines in (**a**), showing the lattice parameter *c* and the projected distances of $In_1$-$In_2$, $Se_1$-$Se_2$, vdW gap, $In_1$-$In_1$ and $Se_1$-$Se_1$.

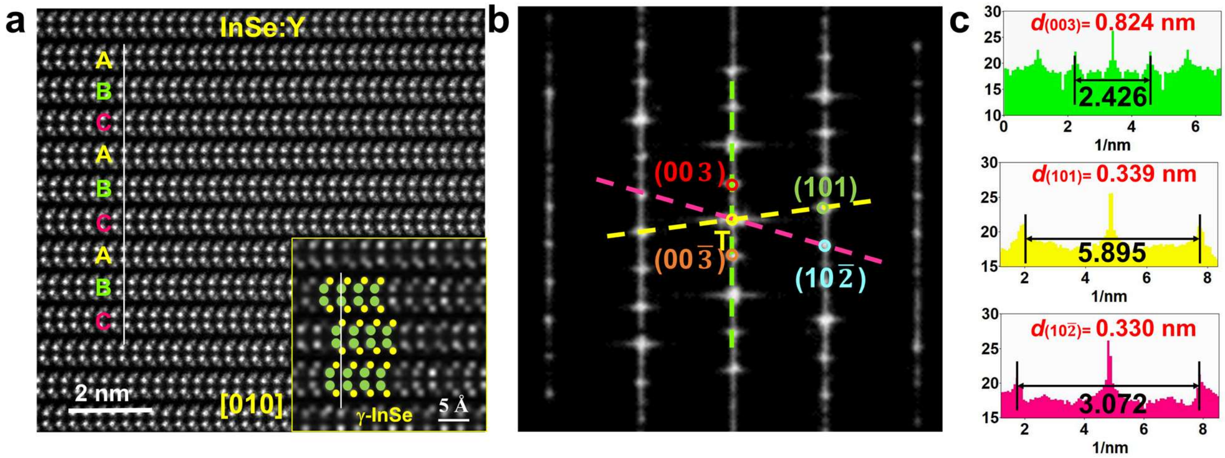


**Figure S12. Further microstructure analyses of InSe:Y. a**, Lower magnified HAADF image projected along [010] zone axis showing the γ-phase-like ABC stacking style along the white line. Inset is the quantitative STEM simulation image of γ-InSe. **b**, The fast Fourier transform (FFT) pattern of Fig. **4a**. **c**, The distance measurements between diffraction points are performed by using the Digital Micrograph software along the green, yellow, pink lines in (**b**), which gives the lattice plane distances of (003), (101) and (10$\bar{2}$).

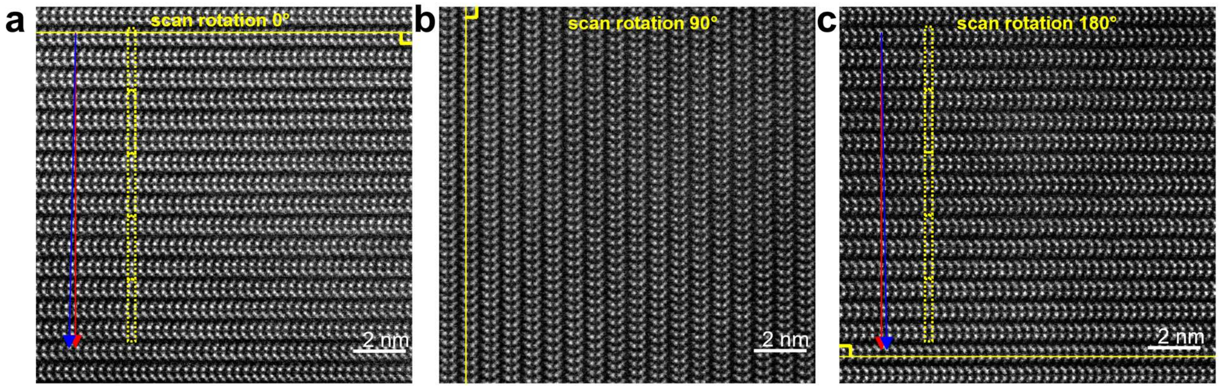


**Figure S13. STEM-HAADF images at different scan rotations. a**, Scan rotation at 0 degree. **b,** Scan rotation at 90 degree. **c,** Scan rotation at 180 degree.

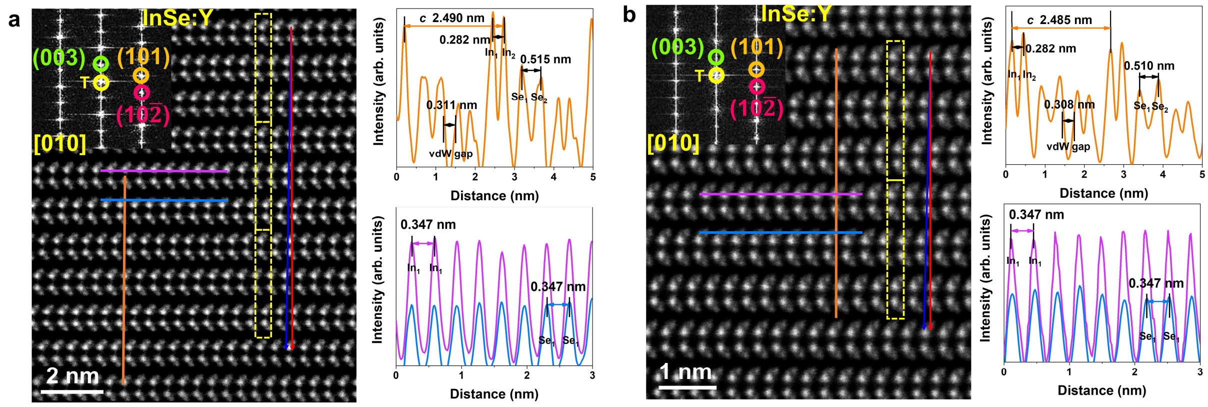


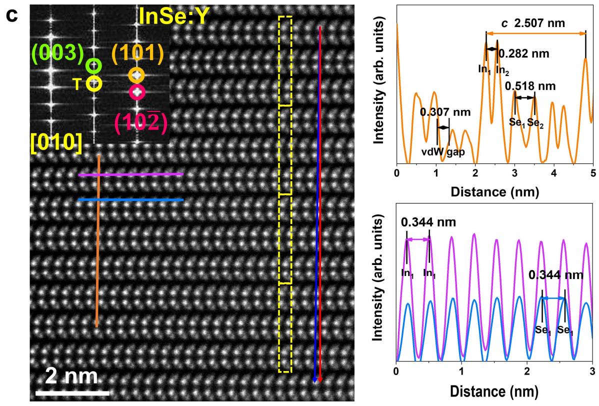


**d**

| *d* / nm | *Sim.* | InSe | InSe:Y | | | |
|---|---|---|---|---|---|---|
| | | | *S1* | *S2* | *S3* | *S4* |
| *c* | 2.532 | 2.530 | 2.473 | 2.490 | 2.485 | 2.507 |
| $d_{(In1-In1)}$ | 0.346 | 0.346 | 0.346 | 0.347 | 0.347 | 0.344 |
| $d_{(Se1-Se1)}$ | 0.346 | 0.346 | 0.346 | 0.347 | 0.347 | 0.344 |
| $d_{(In1-In2)}$ | 0.281 | 0.281 | 0.281 | 0.282 | 0.282 | 0.282 |
| $d_{(Se1-Se2)}$ | 0.536 | 0.535 | 0.515 | 0.515 | 0.510 | 0.518 |
| $d_{(vdW)}$ | 0.308 | 0.310 | 0.308 | 0.311 | 0.308 | 0.307 |

**Figure S14. Further microstructure analysis of different InSe:Y cross-section lamellas. a, b, c,** HAADF images projected along the [010] zone axis with insets of the corresponding FFT patterns (**left**); and the intensity line profiles (**right**) along the purple, sky-blue and orange in left images, showing the lattice parameter *c* and the projected distances of $In_1$-$In_2$, $Se_1$-$Se_2$, vdW gap, $In_1$-$In_1$ and $Se_1$-$Se_1$. The yellow dotted rectangles mark the γ-InSe unit cells. The blue arrows trace the sliding of layers. **d**, Simulated (*Sim.*) and experimental results of *c*-values and distances *d* of In-In, Se-Se and vdW gap for InSe and different InSe:Y (*S1* – *S4*). *S1* is the same sample in Fig. **4a**, and *S2* - *S4* correspond to the samples in (**a** – **c**), respectively.

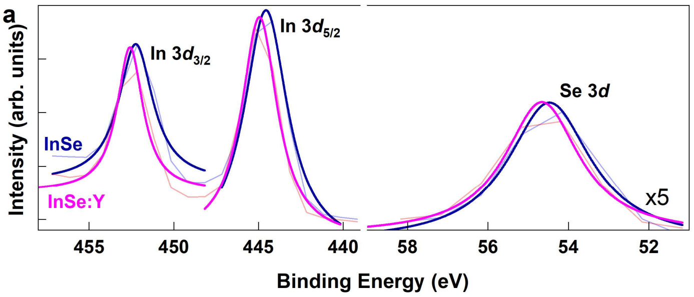


**b**

| XPS peaks | Binding energy (eV) | | Shift (eV) |
|---|---|---|---|
| | InSe | InSe:Y | |
| Se 3*d* | 54.5 | 54.8 | +0.3 |
| In $3d_{5/2}$ | 444.5 | 445.1 | +0.6 |
| In $3d_{3/2}$ | 452.2 | 452.8 | +0.6 |

**Figure S15. XPS results. a**, The XPS spectra of Se 3*d* and In 3*d* of InSe and InSe:Y calibrated with C1*s* at the binding energy of 284.8 eV. **b**, The blue-shift of the peak energy of InSe:Y, as compared with that of InSe.

| 2D materials | Status | Thickness (nm) | $d_{33}^{eff}$ (pm/V) | References |
|---|---|---|---|---|
| 3*R*-like $MoS_2/WS_2$ | Bilayer | | 1.05 | *(26)* |
| 2*H*-like $MoS_2/WS_2$ | Bilayer | | 0.98 | |
| $\alpha$-$In_2Se_3$ | Monolayer | | 0.17 | *(27)* |
| | Bilayer | | 0.30 | |
| | Bulk | 90 | 2.80 | |
| CdS | Ultrathin | 3 | 16.40 | *(28)* |
| | Bulk | | 4.855 | |
| 3*R* $MoS_2$ | Bulk | 18 | 0.45 | *(29)* |
| $\gamma$-InSe | ~Bilayer | 4 | 15.10 | *(30)** |
| | Bulk | 95 | 0.70 | |
| | 48 unit cells | 120 | 4.04 | This work |
| | 20 unit cells | 50 | 7.50 | |
| | 7 unit cells | 18 | 13.32 | |
| | 5 unit cells | 14 | 14.14 | |

**Table S1. Comparison of the measured effective piezoelectric constant $d_{33}^{eff}$ with other 2D materials.** Note: some values from literatures have been conversed by combining with the effective piezoelectric constant in the weak-indentation limit, $d_{33}^{eff}$, exhibiting a good correlation with $d_{33}$, i.e., $d_{33}^{eff}$=0.5$d_{33}$; ii) *The $d_{33}^{eff}$ obtained in this literature is based on the suspension method, which should be much larger than that obtained on a flat substrate. For instance, the literature also pointed out that a value of ~7.5 pm/V was obtained for monolayer $MoS_2$ with the suspension method, but only ~1.12 pm/V on a flat substrate.

## Supplementary References


1. Wei, T. R. et al. Exceptional plasticity in the bulk single-crystalline van der Waals semiconductor InSe. *Science* **369**, 542-545 (2020).
2. Sang, D. K. et al. Two dimensional β-InSe with layer-dependent properties: band alignment, work function and optical properties. *Nanomater.* **9**, 82 (2019).
3. Hu, H. et al. Room-temperature out-of-plane and in-plane ferroelectricity of two-dimensional β-InSe nanoflakes. *Appl. Phys. Lett.* **114**, 252903 (2019).
4. Hu, H. W. et al. Out-of-plane and in-plane ferroelectricity of atom-thick two-dimensional InSe. *Nanotech.* **32**, 385202 (2021).
5. Hao, Q. et al. Phase identification and strong second harmonic generation in pure ε-InSe and its alloys. *Nano Lett.* **19**, 2634-2640 (2019).
6. Hopkinson, D. G. et al. Formation and healing of defects in atomically thin GaSe and InSe. *ACS Nano* **13**, 5112-5123 (2019).
7. Andersen, T. I. et al. Excitons in a reconstructed moiré potential in twisted $WSe_2$/$WSe_2$ homobilayers. *Nat. Mater.* **20**, 480-487 (2021).
8. Sui, F. et al. Atomic insights into the influence of Bi doping on the optical properties of two-dimensional van der Waals layered InSe. *J. Phys. Condens. Mat.* **34**, 224006 (2022).
9. Zhang, B. et al. Super deformability and thermal electricity of bulk γ-InSe single crystals. *Chin. Phys. B* **30**, 078101 (2021).
10. Tauc, J., Grigorovici, R. & Vancu, A. Optical properties and electronic structure of amorphous germanium. *phys. stat. sol.* **15**, 627 (1966).
11. Singh, H. et al. Composition dependent structural phase transition and optical band gap tuning in InSe thin films. *Heliyon* **5**, e02933 (2019).
12. Parlak, M. & Ercelebi C. The effect of substrate and post-annealing temperature on the structural and optical properties of polycrystalline InSe thin films. *Thin Solid Films* **322**, 334-339 (1998).
13. Segura, A., Pomer, F., Cantarero, A., Krause, W. & Chevy, A. Electron scattering mechanisms in n-type indium selenide. *Phys. Rev. B* **29**, 5708-5717 (1984).
14. Tran, K. et al. Evidence for moiré excitons in van der Waals heterostructures. *Nature* **567**, 71-75 (2019).

15. Seyler, K. L. et al. Signatures of moiré-trapped valley excitons in $MoSe_2/WSe_2$ heterobilayers. *Nature* **567**, 66-70 (2019).

16. Guan, Z. et al. Identifying intrinsic ferroelectricity of thin film with piezoresponse force microscopy. *AIP Adv.* **7**, 095116 (2017).

17. Kim, B., Seol, D., Lee, S., Lee, H. N. & Kim, Y. Ferroelectric-like hysteresis loop originated from non-ferroelectric effects. *Appl. Phys. Lett.* **109**, 102901 (2016).

18. Vasudevan, R. K. Balke, N. Maksymovych, P. Jesse & S. Kalinin S. V. Ferroelectric or non-ferroelectric: Why so many materials exhibit "ferroelectricity" on the nanoscale. *Appl. Phys. Rev.* **4**, 021302 (2017).

19. Zeng, Q. et al. Nanoscale ferroelectric characterization with heterodyne megasonic piezoresponse force microscopy. *Adv. Sci.* **8**, 2003993 (2021).

20. Seol, D. et al. Determination of ferroelectric contributions to electromechanical response by frequency dependent piezoresponse force microscopy. *Sci. Rep.* **6**, 30579 (2016).

21. Kang, S. et al. Tunable out-of-plane piezoelectricity in thin-layered $MoTe_2$ by surface corrugation-mediated flexoelectricity. *ACS Appl. Mater. Inter.* **10**, 27424-27431 (2018).

22. Nellist, P. D. & Pennycook, S. J. Subangstrom resolution by underfocused incoherent transmission electron microscopy. *Phys. Rev. Lett.* **81**, 4156-4159 (1998).

23. Yang, L. et al. Chloride molecular doping technique on 2D materials: $WS_2$ and $MoS_2$. *Nano Lett.* **14**, 6275-6280 (2014).

24. Li, F. et al. Giant piezoelectricity of Sm-doped $Pb(Mg_{1/3}Nb_{2/3})O_3$-$PbTiO_3$ single crystals. *Science* **364**, 264-268 (2019).

25. Zheng, B. et al. Rare-earth doping in nanostructured inorganic materials. *Chem. Rev.* **122**, 5519-5603 (2022).

26. Rogée, L. et al. Ferroelectricity in untwisted heterobilayers of transition metal dichalcogenides. *Science* **376**, 973–978 (2022).

27. Xue, F. et al. Multidirection piezoelectricity in mono- and multilayered hexagonal $\alpha$-$In_2Se_3$. *ACS Nano* **12**, 4976-4983 (2018).

28. Wang, X. et al. Subatomic deformation driven by vertical piezoelectricity from CdS ultrathin films, *Sci. Adv.* **2**, e1600209 (2016).

29. Hallil, H. et al. Strong piezoelectricity in 3R-$MoS_2$ flakes. *Adv. Electron. Mater.* **2101131** (2022).

30. Wang, X. et al. Probing effective out-of-plane piezoelectricity in van der Waals layered materials induced by flexoelectricity. *Small* **15**, e1904116 (2019).
